# *EMC6/TMEM93* suppresses glioblastoma proliferation by modulating autophagy

**DOI:** 10.1038/cddis.2015.408

**Published:** 2016-01-14

**Authors:** X Shen, S Kan, J Hu, M Li, G Lu, M Zhang, S Zhang, Y Hou, Y Chen, Y Bai

**Affiliations:** 1 Department of Cell Biology, School of Basic Medical Sciences, Peking University Health Science Center, Beijing, China; 2 Key Laboratory of Medical Immunology, Ministry of Health, Peking University Health Science Center, Beijing, China; 3 Peking University Center for Human Disease Genomics, Peking University, Beijing, China; 4 Department of Physiology, Yong Loo Lin School of Medicine, National University of Singapore, Singapore, Singapore; 5 Department of Cardiology, Peking University Third Hospital, Beijing, China

## Abstract

EMC6 (endoplasmic reticulum membrane protein complex subunit 6), also known as transmembrane protein 93, is a novel positive autophagy regulator. In this report, we evaluated the anti-tumor activity of EMC6 in glioblastoma cells *in vitro* and *in vivo*. Our data show that overexpression of EMC6 in three glioblastoma cell lines (SHG44, U87 and U251) suppresses tumor cell growth by activating autophagy, but fails to induce cell apoptosis. EMC6-mediated autophagy was associated with inactivation of the PIK3CA/AKT/mTOR signaling pathway. Accordingly, *EMC6* knockdown in glioblastoma cells had the opposite effect; it promoted cell growth. Overexpression of EMC6 also sensitized glioblastoma cells to the chemotherapy drug, temozolomide, to further suppress tumor growth. Our data indicate that EMC6-induced autophagy may play a positive role in suppressing the development of glioblastoma.

Glioblastoma (GBM) is one of the most common, aggressive and malignant brain tumors in the central nervous system.^1^ While there have been considerable advances in the multimodality treatment of GBM in the last few decades, only minimal improvements in the median survival time and the 5-year survival rate occurred.^2^ Therefore, uncovering the tumorigenesis mechanism of GBM is essential for finding novel treatments to improve patient prognosis.

Macroautophagy (hereafter called autophagy) is an evolutionarily conserved process in which cellular proteins and organelles are engulfed by autophagosomes and eventually delivered to lysosomes for degradation.^3, 4^ It occurs in a variety of cell types and is associated with cell survival and cell death by regulating intracellular metabolism.^5^ Autophagy is an effective way to degrade aged or malfunctioning organelles and damaged or misfolded proteins, to maintain cellular homeostasis and genomic integrity.^6^ While dysregulation of autophagy is associated with malignant transformation and the suppression of tumorigenesis,^7, 8, 9^ its role in GBM remains unclear.

In GBM cells, cytoplasmic mRNA and protein levels of autophagosome markers (e.g., Beclin-1 and microtubule-associated protein light chain 3 (LC3)) are lower than in normal brain tissue.^10, 11, 12, 13, 14^ This becomes more evident in higher grade GBM, suggesting that the autophagy level is decreased in these cases.^15, 16, 17, 18^ In addition, malignant GBM cells treated with *γ*-irradiation or chemotherapeutic agents, such as temozolomide (TMZ) and arsenic trioxide, have a tendency to undergo autophagy.^19, 20, 21, 22^ This suggests that autophagy plays a role in suppressing the growth of GBM. However, the precise mechanism through which autophagy regulates GBM remains largely unknown, and the exact molecular targets are yet to be identified.

*EMC6* (ER membrane protein complex subunit 6), also known as transmembrane protein 93 (TMEM93), is an autophagy-related gene located on chromosome 17p13.2.^23^ *EMC6* is conserved in cow, mouse, chicken, zebrafish and xenopus. *Homo sapiens* and *Mus musculus* share 100% sequence homology for *EMC6*.^23^ The EMC6 protein is located in the outer membrane of the endoplasmic reticulum (ER).^23^ Expression profile analysis indicated that *EMC6* mRNA has been found in a variety of normal human tissues, including brain, pancreas, kidney, heart, liver, spleen, skeletal muscle and so on.^23^ Compared with these normal tissues, a lower level of EMC6 protein expression was found in a series of cancer tissues, including brain, esophageal and rectal carcinomas, among others (http://www.proteinatlas.org/ENSG00000127774-EMC6/tissue).

We previously showed that EMC6 interacts with the Ras-related protein RAB5A and Beclin-1, and colocalizes with the omegasome marker Zinc finger FYVE domain-containing protein 1 (ZFYVE1) to regulate autophagosome formation in an osteosarcoma cell line.^23^ However, the precise mechanism through which EMC6 regulates the viability of tumor cells, especially GBM cells, remains largely unknown.

In the present study, we observed that overexpression of EMC6 could suppress cell proliferation in three selected GBM cell lines, while knockdown of *EMC6* promoted GBM cell proliferation. Since EMC6 is an autophagy-related protein, we hypothesized that the inhibition of GBM cell proliferation caused by EMC6 overexpression may be related to autophagy. Indeed, we found that EMC6 enhanced the autophagy level in GBM cells by downregulating the phosphatidylinositol 3-kinase (PIK3CA)/protein kinase B (AKT) and the mammalian target of rapamycin (mTOR) pathways. Furthermore, overexpression of EMC6 sensitized GBM cells to TMZ treatment and inhibited GBM formation *in vivo*. This study highlights a novel role of EMC6 in autophagy, which in turn, suppresses GBM cell growth.

## Results

### Overexpression of EMC6 suppresses GBM cell growth

**Figure 1:**
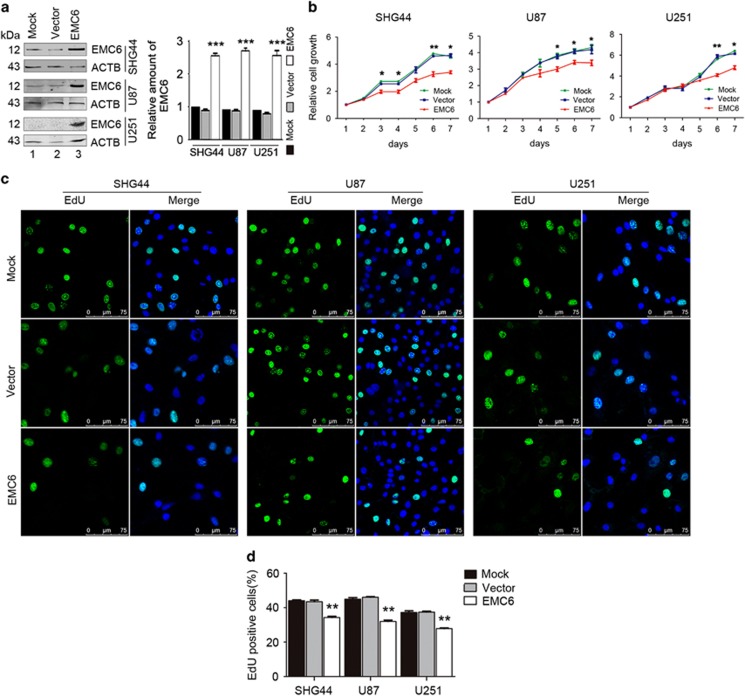
Overexpression of EMC6 suppresses GBM cells growth. (**a**) Expression of EMC6 in GBM cell lines was analyzed by western blot. ACTB was detected as the protein-loading control and quantification of EMC6 levels relative to ACTB in cells treated as in (**a**), ***P<0.001. (**b**) Cell growth of SHG44, U87 and U251 cell lines with or without of EMC6 overexpression was detected by MTS assay. Data were mean±S.D. from three independent experiments. *P<0.05, **P<0.01. (**c**) Cells were plated in glass slides and incorporated with EdU. After 4 h, the indicated cells were performed by immunofluorescence assay. Nuclei were stained with Hoechst 33342. Representative confocal microscopy images were shown. Scale bar, 25 *μ*m. (**d**) Quantification of the percentage of EdU-positive cells in total indicated cells were shown from five randomly selected areas from each slide. Each bar represents the mean±S.D. from three independent experiments. **P<0.01

To study the influence of EMC6 on GBM cell proliferation, we established three GBM cell lines (SHG44, U87 and U251) that stably overexpressed EMC6 (Figure 1a). Next, we performed MTS assays to assess the cell viability in these three EMC6-overexpressing cell lines. Cell viability was significantly reduced in all three EMC6-overexpressing cell lines compared with controls (Figure 1b).

We also measured the level of 5-ethynyl-2′-deoxyuridine (EdU) incorporation in the GBM cell lines, and found that the percentage of EdU-positive cells (i.e., proliferative cells) was significantly decreased in EMC6-overexpressing SHG44 cells (from ~44 to 34%), U87 cells (from ~45 to 32%) and U251 cells (from ~37 to 27%) (Figures 1c and d). These results suggest that overexpression of EMC6 could cause growth arrest of GBM cells.

As apoptosis can affect cell viability, we detected the exposure of phosphatidylserine (PS), which is a key biochemical hallmark of cell apoptosis. We analyzed the percentage of cells undergoing apoptosis in the three GBM cell lines by flow cytometry using FITC-labeled Annexin V. Our data revealed no significant difference in cell apoptosis between the EMC6-overexpressing cells and the control cells (Supplementary Figure 1). Together, these data suggest that overexpression of EMC6 suppresses the growth of GBM cells, but fails to induce cell apoptosis.

### Knockdown of EMC6 promotes GBM cells proliferation

**Figure 2:**
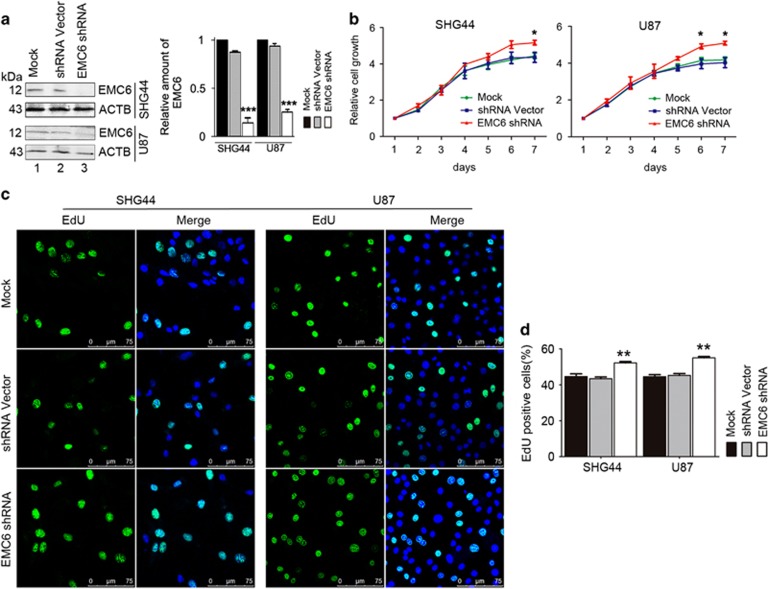
Knockdown of EMC6 induces GBM cells growth. (**a**) Expression of EMC6 in GBM cell lines was analyzed by western blot. ACTB was detected as the protein-loading control and quantification of EMC6 levels relative to ACTB in cells treated as in (**a**), ***P<0.001. (**b**) Cell growth of SHG44, U87 with or without EMC6 knockdown was detected by MTS assay. Data were mean±S.D. from three independent experiments. *P<0.05. (**c**) Cells were plated in glass slides and incorporated with EdU. After 4 h, the indicated cells were performed by immunofluorescence assay. Nuclei were stained with Hoechst 33342. Representative confocal microscopy images were shown. Scale bar, 25 *μ*m (**d**) Quantification of the percentage of EdU-positive cells in total indicated cells were shown from five randomly selected areas from each slide. Each bar represents the mean±S.D. from three independent experiments. **P<0.01

To further confirm the function of EMC6 in GBM cells, we established stable *EMC6*-knockdown cell lines. EMC6 expression was almost absent in SHG44 and U87 cell lines (Figure 2a). However, we did not establish a stable *EMC6*-knockdown U251 line, as EMC6 expression was already too low to be observed by western blot in the mock-transfected U251 cells (Figure 1a). Cell viability was significantly elevated in *EMC6*-knockdown cells compared with the controls (Figure 2b). In addition, after *EMC6*-knockdown, the percentage of EdU-positive cells (proliferative cells) were higher than in the control cells, that is, from 44 to 53% in SHG44 cells and from 45 to 56% in U87 cells (Figures 2c and d). No apoptosis in *EMC6*-knockdown cells was detected (Supplementary Figure 2). Collectively, our data from the *EMC6*-knockdown cells confirms that EMC6 suppresses GBM cell proliferation, but does not induce apoptosis.

### EMC6 promotes autophagic flux in GBM cells

**Figure 3:**
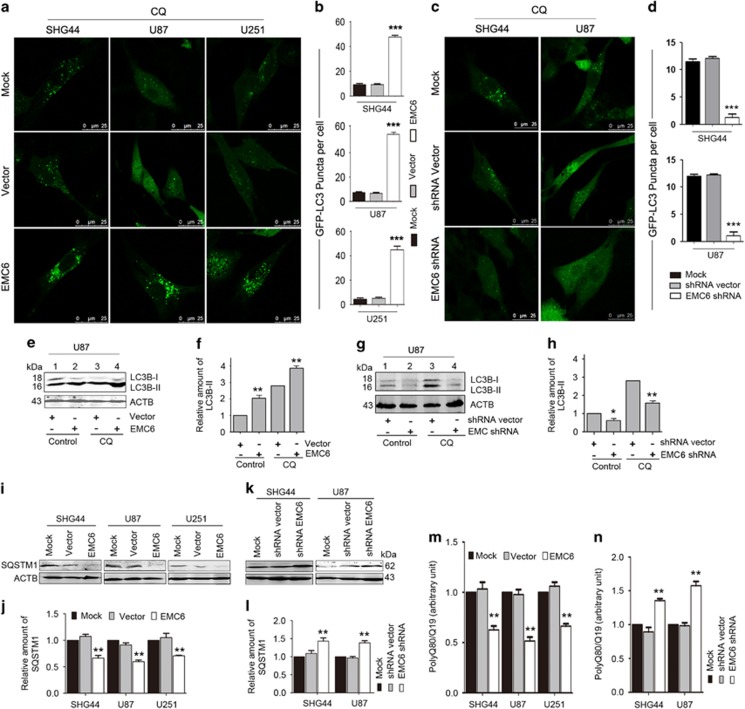
EMC6 promotes autophagic flux in GBM cells. (**a** and **c**) The indicated cell lines with or without EMC6 overexpression (or EMC6 knockdown) were infected with Ad5–GFP–LC3 at 50 MOI for 20 h, then treated in the presence or absence of CQ (25 *μ*M) for 2 h. Representative confocal microscopy images of GFP–LC3B distribution obtained from the indicated cell lines are shown (**b** and **d**). Quantification of GFP–LC3B puncta per cell treated as in (**a**) and (**c**). Data are means±S.D. of at least 100 cells scored. ***P<0.001. (**e** and **g**) U87 cells with or without EMC6 overexpression (or EMC6 knockdown) were cultured and treated in the presence or absence of CQ (25 *μ*M) for 2 h. The levels of LC3B-II were detected by western blot. (**f** and **h**) Quantification of LC3B-II levels relative to ACTB in cells treated as (**e**) and (**g**). Average value in vector group without CQ treatment was normalized as 1. Data are means±S.D. of results from three experiments, *P<0.05, **P<0.01. (**i** and **k**) The levels of SQSTM1 in the indicated cell lines with or without EMC6 overexpression (or EMC6 knockdown) were analyzed by western blot. (**j** and **l**) Quantification of amounts of SQSTM1 relative to ACTB in cells treated as in (**i**) and (**k**). The average value in vector group was normalized as 1. Data are means±S.D. of results from three experiments, **P<0.01. (**m** and **n**) The indicated cells were electrotransfected with polyQ80-Firefly luciferase (or control polyQ19-Firefly luciferase) for 24 h. The luminescent signals were detected and polyQ80-luciferase/polyQ19-luciferase ratios were calculated in each group. Data are means±S.D. of results from three experiments, **P<0.01

Next, we evaluated the effect of EMC6 on autophagy activity in GBM cells by measuring the LC3 isoform B (LC3B) autophagy marker. Our confocal microscopy data revealed that EMC6 overexpression increased the puncta distribution of GFP–LC3B compared with controls. Since GFP–LC3B puncta correlate with the number of autophagosomes, our results suggests that EMC6 overexpression either increases autophagosome formation or blocks autophagosome clearance by the lysosomal compartment. Therefore, to analyze the autophagic status in these EMC6-overexpressing cells, we cultured the cells in the presence or absence of chloroquine (CQ), which prevents the fusion between autophagosomes and lysosomes by raising lysosomal pH. We observed that CQ treatment resulted in more GFP–LC3B puncta in EMC6-transfected cells than in the control cells (Figures 3a and b). Next, we measured the LC3B conversion to LC3B-II by western blot. We observed that the overexpression of EMC6 in U87 cells elevated the levels of endogenous LC3B-II with or without CQ (Figures 3e and f). The results in the SHG44 and U251 cell lines were similar to those in the U87 cell line (Supplementary Figures 3a–d), indicating that overexpression of EMC6-induced autophagosome formation.

Subsequent experiments were conducted in *EMC6*-knockdown GBM cell lines. As shown in Figures 3c and d, the distribution of GFP–LC3B puncta was decreased compared with that of control cells in the presence of CQ. Western blot analysis also showed that the LC3B-II was significantly decreased in *EMC6*-knockdown U87 cells with or without CQ treatment (Figures 3g and h). The results in the SHG44 cell line were similar to those in the U87 cell line (Supplementary Figures 3e and h), indicating that knockdown of *EMC6* attenuates autophagosome synthesis.

Accumulating data show that the effect of the GFP–LC3B fusion protein is similar to the endogenous LC3B protein in autophagy. Since GFP is relatively resistant to lysosomal hydrolysis compared with LC3B, the levels of free GFP detected by western blot have been used to measure functional autophagic flux. After cells were infected with Ad5–GFP–LC3B for 24 h, we found that the free GFP band detected by western blot was stronger in EMC6-overexpressing GBM cells than in control cells (Supplementary Figures 3i and j, lane 4 *versus* lane 3). Meanwhile, free GFP was decreased in *EMC6*-knockdown cells compared with the control cells (Supplementary Figures 3i and j, lane 2 *versus* lane 1).

We further performed a time course experiment to determine the levels of LC3B-II and number of cell apoptosis in U87 cells after EMC6 overexpression. Data from western blotting show that the accumulation of LC3B-II was increased in cells after EMC6 overexpression for 12 h, further increased at 24 h and maintained at 36 h (Supplementary Figures 3k and l). Simultaneously, analysis of flow cytometry suggested that there was no change in cell apoptosis of U87 cells after EMC6 overexpression at different time points (Supplementary Figures 3m and n).

In addition, sequestosome-1 (SQSTM1) is widely recognized as a link between LC3 and ubiquitinated substrates, and is also used as a marker for detecting autophagic flux. Consistent with our above observation, we showed that SQSTM1 degradation was enhanced in EMC6-overexpressing GBM cell lines (Figures 3i and j), and was inhibited in *EMC6*-knockdown cell lines (Figures 3k and l). Likewise, overexpression of EMC6 increased the degradation of polyQ80 aggregates formed by a stretch of 80 glutamine residues that is also an autophagic substrate (Figure 3m), indicating that EMC6 overexpression increases the clearance of autophagic substrates. By contrast, the degradation of polyQ80 aggregates protein was impaired in *EMC6*-knockdown GBM cell lines (Figure 3n). Collectively, these results suggest that EMC6 promotes autophagic flux in GBM cell lines.

We also got three chemically synthesized EMC6 siRNAs and found only *siEMC6-3* can significantly decrease the expression of EMC6 (Supplementary Figures 4a and c). Therefore, *siEMC6-3* was selected to repeat the key experiments. We found that the percentage of EdU-positive cells was increased in *siEMC6-3*-transfected U87 cells (Supplementary Figure 4d). Studies on the phenotype of autophagy indicated that *siEMC6-3* attenuated the levels of LC3B-II and increased the accumulation of SQSTM1 (Supplementary Figures 4e and f). These results were as same as that *shEMC6* treatment.

### EMC6 induces autophagy through the PIK3CA/AKT/mTOR pathway

The mTOR plays a central role in the regulation of cell proliferation, growth, differentiation and survival.^24^ Dysregulation of this pathway is closely associated with a wide variety of cancers, including malignant GBMs.^25, 26, 27^ In addition, this pathway is also a canonical pathway that regulates autophagy in cells.^28, 29^ We determined whether autophagy impairment in GBM cells is a result of aberrant mTOR signaling.

**Figure 4:**
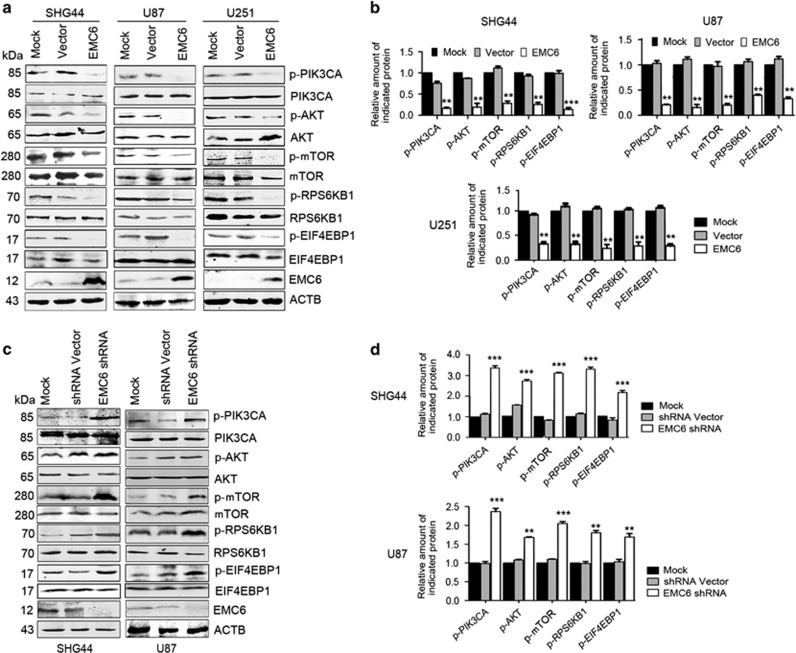
EMC6 modulates autophagy through the PIK3CA/AKT/mTOR signaling. (**a**) The indicated cells with or without EMC6 overexpression were lysed and subjected to immunoblot analysis using the indicated antibodies. The total form of the indicated protein was detected as the protein-loading control. (**b**) Histogram shows the quantification of the ratio of the relative proteins/the total form of (**a**). The average value in mock cells was normalized as 1. Data are means±S.D. of results from three independent experiments. **P<0.01, ***P<0.001. (**c**) The indicated cells with or without EMC6 knockdown were lysed and subjected to immunoblot analysis using the indicated antibodies. The total form of the indicated protein was detected as the protein-loading control. (**d**) Histogram shows the quantification of the ratio of the relative proteins/the total form of (**c**). The average value in mock cells was normalized as 1. Data are means±S.D. of results from three independent experiments. **P<0.01, ***P<0.001

Western blot analysis showed dramatically decreased phosphorylation levels of mTOR (Ser2448) and an mTOR substrate (ribosomal protein 70 S6 kinase or ribosomal protein S6 kinases, polypeptide 1 (RPS6KB1)) in EMC6-overexpressing GBM cells (Figures 4a and b). Similarly, downregulation of phosphorylated eukaryotic translation initiation factor 4E-binding protein 1 (EIF4EBP1), another downstream effector of mTOR, was also observed in EMC6-overexpressing GBM cells (Figures 4a and b). Collectively, these results suggest that EMC6 induces autophagy via inactivation of the mTOR pathway.

The PIK3CA/AKT signaling pathway, which is upstream of the mTOR pathway, is also required for several vesicular trafficking processes.^28^ Therefore, we also analyzed the influence of EMC6 on the activation of the PIK3CA/AKT pathway. We found a significant decrease in the phosphorylation of PIK3CAp85 and AKT (Ser 308) in EMC6-overexpressing GBM cells (Figures 4a and b). Consistent with this, the levels of p-PIK3CA, p-AKT, p-mTOR, p-RPS6KB1 and p-EIF4EBP1 were elevated in the EMC6-knockdown GBM cells (Figures 4c and d).

To further demonstrate whether inhibition of mTOR signaling is necessary for EMC6-mediated autophagy, a constitutively active form of RHEBQ64L, which stimulates the phosphorylation of RPS6KB1 through activation of mTOR signaling, was transfected into U87 cells to recover mTOR activity. As shown in Supplementary Figures 5a and b, the phosphorylation level of RPS6KB1 was upregulated in RHEBQ64L-overexpressing cells, indicating that the activity of mTOR was restored in EMC6-overexpressing U87 cells. Simultaneously, overexpression of RHEBQ64L increased the SQSTM1 levels and decreased LC3B-II accumulation induced by EMC6 (Supplementary Figures 5a and b, lane 4 *versus* lane 2). Conversely, overexpression of an inactive form of RHEBD60K failed to reverse the activity of mTOR and the level of autophagy (Supplementary Figures 5a and b, lane 6 *versus* lane 2). The results indicate that inhibition of mTOR signaling contributes to EMC6-induced autophagy.

We also showed that treatment with perifosine, an AKT inhibitor, inhibited the phosphorylation of AKT in U87 cells (Supplementary Figures 5c and d). At the same time, perofosine increased the LC3B-II levels in *EMC6*-knockdown cells (lane 4 *versus* lane 3). These results suggested that EMC6-mediated autophagy is involved with the AKT activity. Taken together, our results indicate that the PI3K/AKT/mTOR signaling pathway is related to EMC6-regulated autophagy.

Our previous report demonstrated that EMC6-mediated autophagy is associated with the activity of class III PI3K in the osteosarcoma U2OS cell line.^23^ Here, we also found that the treatment of 3-methyladenine (3-MA, an inhibitor of class III PI3K) could attenuate the LC3B-II accumulation in U87 cells induced by EMC6 overexpression (Supplementary Figures 5e and f). These results indicate that the activity of class III PI3K is at least partly involved with EMC6-mediated autophagy.

As EMC6 is located in the ER, we examined whether the EMC6-induced autophagy is associated with ER stress. Using real-time PCR, we found no difference in the expression levels of *ATF4*, *CHOP*, *ATF6*, *GRP78*, *ATG5* and *ATG6* between EMC6-overexpressing, *EMC6*-knockdown cells and control cells (Supplementary Figures 6a and e). This suggests that EMC6-mediated autophagy is independent of ER stress. Collectively, these data suggest that EMC6 induces autophagy via inactivation of the PIK3CA/AKT/mTOR signaling and PIK3C3 activity.

### Overexpression of EMC6 sensitizes GBM cell lines to chemotherapy

**Figure 5:**
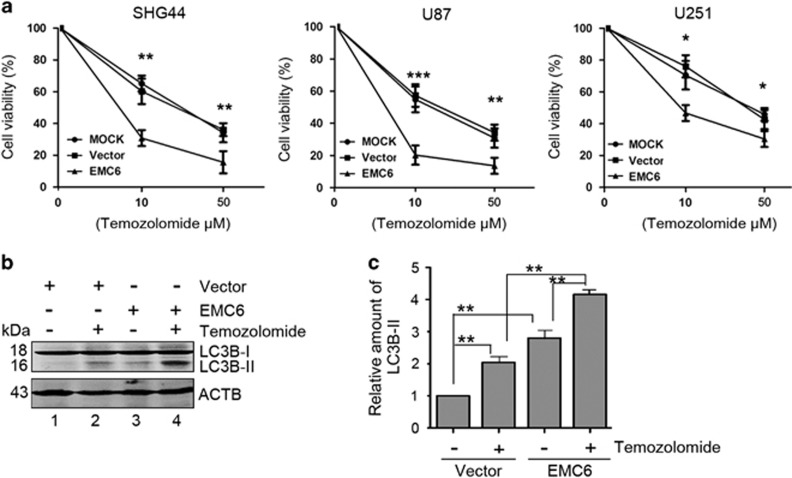
Overexpression of EMC6 sensitizes GBM cell lines to chemotherapy. (**a**) The indicated cells with or without EMC6 overexpression were cultured in 96-well noncoated plate with 5000 cells per well and in 5 replicate wells. After plating, three group cells were managed with 0, 10, 50 *μ*M temozolomide for 48 h and their percentages of cell viability were detected by MTS assay. The GBM cells sensitivity to TMZ were shown by plotting diagram. Data are means±S.D. of results from three independent experiments. *P<0.05, **P<0.01, ***P<0.001. (**b**) The levels of LC3B-II in the U87 cell treated with temozolomide or/and EMC6 overexpression were analyzed by western blot. (**c**) Histogram shows the quantification of the ratio of the LC3B-II/ACTB in (**b**). The average value in control group was normalized as 1. Data are means±S.D. of results from three independent experiments. **P<0.01

TMZ, a first-line drug used in GBM treatment, is able to reduce viability and induce G2/M arrest in GBM cells.^30^ TMZ was also reported to induce autophagy. Therefore, it is possible that overexpression of EMC6 can also enhance the sensitivity of GBM cells to chemotherapy by inducing autophagy. To this end, we treated the aforementioned GBM stable cell lines with 0, 10, 50 *μ*M of TMZ for 48 h and determined cell viability by MTS assays. As expected, TMZ treatment inhibited the cell viability of GBM cells in a dose-dependent manner. Moreover, this suppression of cell viability was enhanced in EMC6-overexpressing GBM cells (Figure 5a), suggesting that EMC6 can enhance the sensitivity of GBM cells to chemotherapy.

In addition, western blot analysis showed that both TMZ treatment and EMC6 overexpression could elevate LC3B-II substantially compared with controls in U87 cells (Figures 5b and c, lane 2 *versus* lane 1, lane 3 *versus* lane 1). TMZ combined with EMC6 further promoted LC3B-II accumulation more than a single treatment of TMZ or EMC6 overexpression alone (Figures 5b and c, lane 4 *versus* lane 2, lane 4 *versus* lane 3). These data suggest that the TMZ-induced inhibition of GBM cell viability is due to the activation of autophagy, which is enhanced by the concomitant overexpression of EMC6.

### EMC6 inhibits the development of GBM *in vivo*

To further verify the anti-tumor properties of EMC6, we detected whether EMC6 could inhibit the intracranial tumor in BALB/c nude mice *in vivo*. Based on our preliminary experiments, we found that the U87 cell line possesses an obvious tumorigenicity compared with other cell lines in our study. Therefore, we only used the U87 cell line for our *in vivo* experiments.

**Figure 6:**
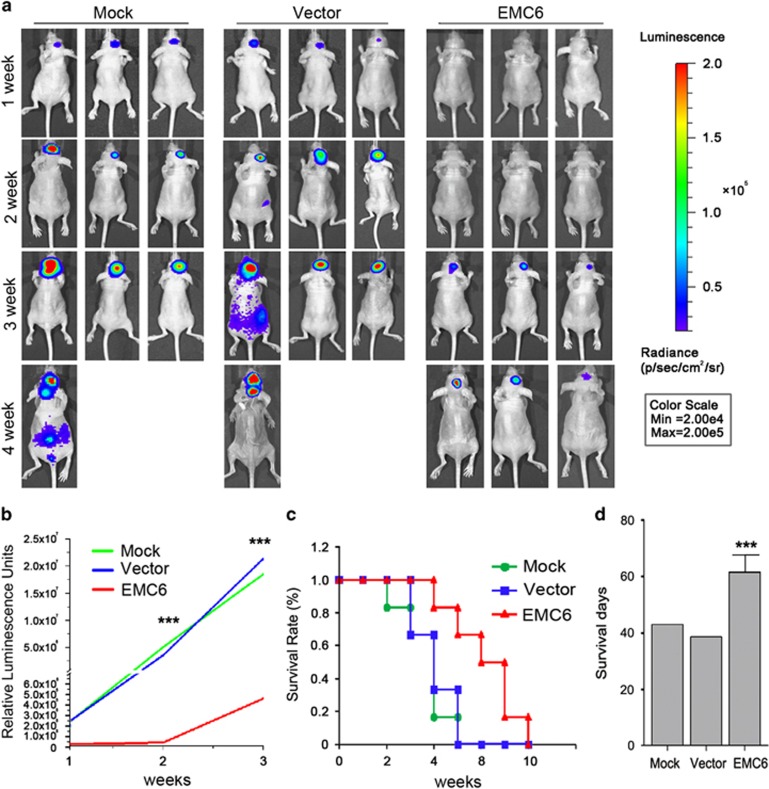
Overexpression of EMC6 inhibits the development of GBM *in vivo*. (**a**) The U87 with or without EMC6-overexpression cells stably expressing luciferase were injected into intracranial brain of BALB/c nude mice. Luciferase signal in different treatment groups was detected every 7 days by *in vivo* bioluminescence imaging (IVIS 100 Imaging System) to monitor the development of GBM until 4 weeks. *n*=6. (**b**) Plotting diagram shows the changes of relative luminescence in the indicated groups of mice during 3 weeks. Data are means±S.D., ***P<0.001. *n*=6. (**c**) Survival differences among the indicated groups of mice were shown by survival curve until all mice were dead. *n*=6. (**d**) The survival day differences among the indicated groups of mice were shown by survival curve until all mice were dead. *n*=6. ***P<0.001

As luciferase is the most convenient and quickest way to detect the location and size of GBM dynamically, we established three stably expressing luciferase U87 cell lines (i.e., mock, null vector and EMC6-overexpressing) and injected them into the brain of nude mice. Using these three groups of intracranial GBM animal models, we imaged the mice using a luciferase reporter assay system on day 7, 14, 21 and 28 after cell seeding. The luminescence signals of nude mice with EMC6 overexpression were significantly smaller than controls (Figures 6a and b). In addition, EMC6 overexpression inhibited GBM progression and prolonged the survival of mice (Figures 6c and d). In contrast, EMC6 knockdown promoted GBM progression and shortened the survival of mice compared with the control group (Supplementary Figures 7a and b).

**Figure 7:**
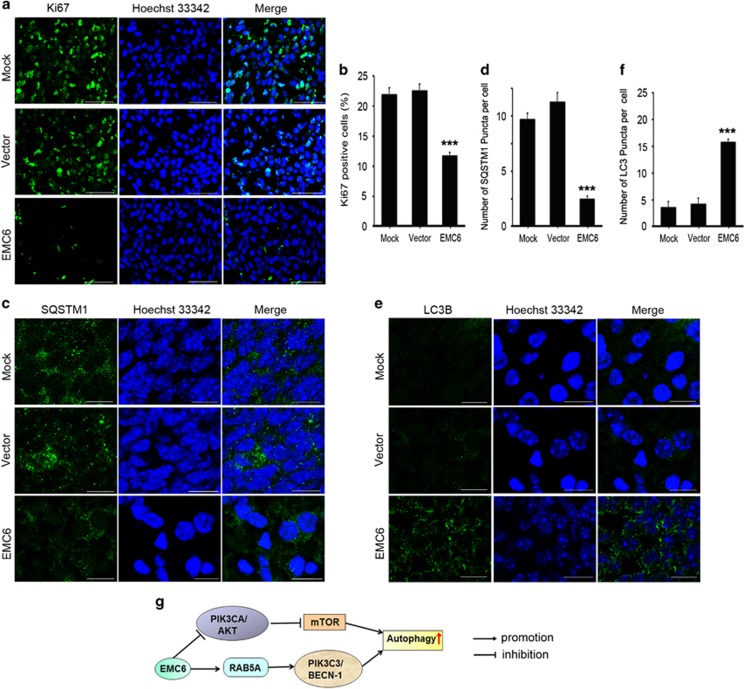
Overexpression of EMC6 inhibits cell proliferation, increases autophagy in GBM. (**a**) The brain frozen sections were obtained from the indicated groups of mice and performed by immunofluorescence assay. Representative confocal microscopy images of the fluorescence of Ki67 were analyzed. Nuclei were stained with Hoechst 33342. Scale bar, 50 *μ*m. (**b**) Five randomly selected areas from each slide were examined for the percentage of Ki67-positive cells in total cells under fluorescence microscope. All experiments were performed in triplicate. Data are means±S.D., ***P<0.001. (**c**) The treatment of frozen sections is same as (**a**). Representative confocal microscopy images of the SQSTM1 distribution were analyzed. Scale bar, 10 *μ*m (**d**) Five randomly selected areas from each slide were examined for the number of SQSTM1 puncta per cell under fluorescence microscope. All experiments were performed in triplicate. Data are means±S.D., ***P<0.001. (**e**) The treatment of frozen sections is same as (**a**) Representative confocal microscopy images of the distribution of LC3B were analyzed. Scale bar, 10 *μ*m (**f**) Five randomly selected areas from each slide were examined for the number of LC3 puncta per cell under fluorescence microscope. All experiments were performed in triplicate. Data are means±S.D., ***P<0.001. (**g**) A schematic model of EMC6-regulating autophagy

To further study the tumor-inhibiting mechanism of EMC6, we made frozen sections of whole brain specimens from the mock, vector and EMC6-overexpressing groups of mice at day 21. Mice at day 21 were chosen as the intracranial implanted GBM had been successfully established in the control group by this time. We first examined the expression of the Ki-67 nuclear antigen in the brain sections. Ki-67 expression varies in different cell cycle phases (i.e., cells express Ki-67 during G1, S, G2 and mitotic phases, but not during the resting phase G0).^31^ A Ki67 staining assay was also used to evaluate cell proliferation.^32^ We found the proportion of Ki-67-positive cells in the *EMC6*-overexpression group was significantly less than that in the null and mock groups (Figures 7a and b). Next, the brain sections were stained to detect apoptosis using the *in situ* nick-end labeling (TUNEL) staining technique.^33^ We found no significant differences among the three groups (data not shown).

To further assess whether tumor suppression was related to autophagy induced by EMC6, we detected LC3B and SQSTM1 by immunofluorescence in the null, mock and EMC6-overexpressing groups of mice. As shown in Figures 7c and d, the signal of SQSTM1 was decreased, and the number of LC3B puncta was increased in EMC6-overexpressing mice compared with the null and mock groups (Figures 7e and f). This indicates that EMC6-induced activation of autophagy may be responsible for the inhibition of GBM formation *in vivo*.

## Discussion

EMC6 is a novel autophagy-related molecule that was first reported by Li *et al.*^23^ In this study, we investigated the mechanism through which EMC6 regulates GBM. We found that EMC6 suppressed the proliferation of GBM cells and improved the sensitivity of GBM cells to TMZ by modulating autophagy. Furthermore, it was found that the PIK3CA/AKT/mTOR pathway was activated on *EMC6* knockdown, and was inhibited by EMC6 overexpression. Finally, EMC6 expression inhibited the xenograft tumor in *in vivo* assays in mice. Together, this suggests that EMC6 can significantly inhibit GBM advancement by stimulating autophagy.

Impairment of autophagy is associated with cancer development. This study also showed that overexpression of EMC6 in GBM cells resulted in increased LC3B-II and reduced SQSTM1, and *EMC6* knockdown had the opposite effect. Elevation of SQSTM1 is considered as a hallmark for impaired autophagy and has been associated with poor prognosis in some tumor types. For example, Burdelski *et al.*^35^ found that high levels of SQSTM1 were significantly linked to increased tumor cell proliferation in prostate cancers patients, indicating that impairment of autophagy is closely correlated with cancer cell proliferation.^34^ In this study, we found that *EMC6* knockdown enhanced GBM cell proliferation, indicating that autophagy can negatively regulate GBM cancer cell proliferation.

Autophagy is mainly regulated by the mTOR pathway, which is a downstream effector of the PIK3CA/AKT signaling pathway. Previous studies demonstrated that the mTOR pathway is associated with cancer pathogenesis.^36^ In addition, many components of the PIK3CA pathway are mutated in human cancers,^37^ suggesting an essential role of the PIK3CA/AKT/mTOR pathway in cancer development. More recently, the mTOR pathway was shown to be a central modulator in cell proliferation of malignant GBMs.^38^ In this study, we found that overexpression of EMC6 inactivated the PIK3CA/AKT/mTOR pathway, while *EMC6* knockdown activated the PIK3CA/AKT/mTOR signaling (Figure 4). Combined with the results of previous studies,^23^ there are two possible mechanisms for EMC6 in autophagy regulation. On the one hand, EMC6 could regulate the recruitment of RAB5A to the ER, then facilitate the interaction between RAB5A and PIK3C3, enhance phosphatidylinositol 3-phosphate production and thereby promote autophagosome formation (Figure 7g). On the other hand, EMC6 may inhibit the PIK3CA/AKT/mTOR pathway in an unknown manner, which requires further investigation.

Besides its role in cancer cell growth and proliferation, the mTOR pathway also plays an important role in cancer formation. Increasing evidence points to deregulation of protein synthesis downstream of mTOR, at the level of EIF4EBP1, as playing a central role in tumor formation.^39^ EIF4EBP1 is capable of mediating the oncogenic effects of AKT signaling on mRNA translation, cell growth and tumor progression.^40^ In this study, we found that overexpression of EMC6 reduces levels of p-EIF4EBP1, while *EMC6* knockdown elevates p-EIF4EBP1 levels. We also showed that overexpression of EMC6 inhibits GBM formation *in vivo*. Therefore, through inactivation of the mTOR pathway, EMC6 reduces the levels of tumor-forming proteins, which subsequently may inhibit GBM formation.

Diseases associated with autophagy defects, including cancer, may be treated by modulating the process of autophagy.^38, 41^ For example, pharmaceuticals such as TMZ, a DNA alkylating agent that can induce both apoptosis and autophagy, have been used to treat malignant GBM cells.^42^ However, high doses of TMZ cause toxicity and side effects.^43^ We found that EMC6 and TMZ can induce autophagy individually, but combining TMZ with EMC6 further enhanced the autophagy level. As EMC6-induced autophagy may enhance the sensitivity of GBM cells to TMZ, we may be able to reduce the required dosage of TMZ and therefore lessen its side effects. Therefore, the use of EMC6 together with chemotherapeutic drugs (e.g., TMZ) may be beneficial in the future for treatment of GBM.

In conclusion, this study shows that EMC6 overexpression suppresses the proliferation of GBM cells by inducing autophagy via downregulating the PIK3CA/AKT/mTOR signaling pathway. Furthermore, overexpression of EMC6 sensitizes GBM cells to TMZ treatment, and inhibits GBM formation *in vivo*. Our investigations provide insight into the activities of EMC6 and its role in inhibiting GBM cell proliferation.

## Materials and Methods

### Antibodies and reagents

The following antibodies were used: rabbit anti-mouse p62/SQSTM1 antibody (MBL International, Japan; PM045), anti-LC3B (Sigma, St Louis, MO, USA; L7543), anti-mTOR (CST, Boston, MA, USA; 7C10, anti-phospho-mTOR (Ser2448; CST; 2971S), anti-RPS6KB1 (CST; 4907), anti-Phospho-RPS6KB1 (Thr389) (CST; 9234s), anti-PIK3CAp85 antibody (CST; 4292), anti Phospho-PIK3CAp85 (Tyr458) (CST; 4228), anti-AKT antibody (CST; 9272s), anti phospho-AKT (Ser473) (CST; 4060s), anti-phospho-EIF4EBP1 (Thr37/46) (CST; 236B4) and anti-EIF4EBP1 (Abcam, Cambridge, UK, ab2606). Secondary antibodies included Alexa Fluor 488-labeled goat anti-mouse and rabbit IgG(H+L) (ZSGB-BIO; ZF-0512 and ZF-0511), Alexa Fluor 594-labeled goat anti-mouse and rabbit IgG(H+L) (ZSGB-BIO; ZF-0513 and ZF-0516), DyLight 680-conjugated anti-mouse and rabbit IgG (H&L) (Rockland, Limerick, PA, USA; 610-144-002 and 611-144-002), DyLight 800-conjugated anti-mouse and Rabbit IgG (H&L) (Rockland; 610-145-002 and 611-145-002). Other reagents used in this study were: Dual-Luciferase Reporter (DLR) Assay System (Promega, Madison, WI, USA; E1910), Hoechst 33342 (Life, Carlsbad, CA, USA; H1399), CQ (Sigma-Aldrich, St Louis, MO, USA; c6628), 3-MA (NSC 66389) and Perifosine (Selleck, Houston, TX, USA, S1037).

### Cell culture and electrotransfections

U251, U87 and SHG44, which are from Institute of Basic Medical of Science, Research Chinese Academy of Medical Sciences, were cultured in MEM (Hyclone, Houston, TX, USA), H-DMEM (Hyclone) and PRIM 1640 (Hyclone) medium, respectively, with 10% fetal bovine serum. All the cells were maintained at 37 °C in a humidified chamber with 5% CO2. SHG44, U87, U251 cells were electrotransfected with pCDB-EMC6, which is the same vector as in the previous study (Li *et al.*^23^) and then their pools were selected by puromycin to establish the EMC6 overexpression GBM cell strains, pCDB-Vector as the control vector.

SHG44, U87 cells were electrotransfected with EMC6 shRNA, which had an excellent knocking down expression of EMC6 chosen from four shRNAs by previous experiment, and selected by puromycin to establish the *EMC6*-knockdown GBM cell strains; shRNA vector as the control vector. Specific shRNA-mediating EMC6 gene knockdown with the targeting sequence: 5′-GCCTCTTCACCTACGTCCTGTTCTGGACG-3′, and nonsilencing shRNA vectors were constructed by ORIGEN Corporation (Austin, TX, USA).

EMC6 overexpression U87 cells and control cells were electrotransfected with luciferase vector and selected by G418 to establish the stably expressing luciferase cell strains.

### Protein extraction and western blotting

Total proteins from cells were extracted with immunoprecipitation assay buffer (50 mM Tris, pH 7.4, 150 mM NaCl, 1% Triton X-100, 1% sodium deoxycholate, 0.1% SDS and with freshly added proteinase inhibitor cocktail and phosphatase inhibitors). Protein concentrations were determined by the BCA protein assay reagent (NCI3227CH, Pierce, Thermo Fisher Scientific, Pittsburgh, PA, USA). Equal amounts of proteins were separated by SDS-PAGE electrophoresis and transferred to nitrocellulose membranes. After blocking with 5% nonfat milk for 1 h at room temperature, the membranes were incubated overnight at 4 °C with primary antibodies and then the secondary antibodies. The membranes were then washed with PBS containing 0.1% Tween 20 and scanned with the Odyssey Infrared Imaging System (LI-COR Biosciences, Lincoln, NE, USA) by setting the detection channels at 700 nm (for Alexa Fluor 680) and 800 nm (for IRDye 800CW, Rockland, Limerick, PA, USA). Scanned bands were quantified using the Image J software and normalized against the protein level of *β*-actin/ACTB. Results are representative of at least three experiments.

### Cells viability detection

Cell viability assays were performed using the CellTiter 96 AQueous One Solution Cell Proliferation Assay (Promega, G3582, USA). According to the manufacturer's protocols, cells were seeded in multiple 96-well plates at 1 × 10^3^ cells/well in 100 *μ*l full growth medium. Four hours before experiment, 10 *μ*l/well CellTiter 96 AQueous One Solution was added to a plate and incubated for 4 h at 37 °C in a humidified, 5% CO2 incubator, then removed and assayed on an EL-311SX ELISA Reader (Bio-Tec Instruments, Winooski, VT, USA) at 490 nm to establish baseline readings. Successive readings were conducted on remaining plates every 24 h from plating to 7 days post seeding to establish growth curves. Cell viability was calculated as follows: cell viability=absorbance of test group/absorbance of control cell group × 100%. Each experiment was performed in three replicate wells and independently repeated four times.

### EdU incorporation detection

Cell proliferation was detected using EdU detection kit (Invitrogen, Carlsbad, CA, USA, C10639). Cells were cultured in common medium and plated in coverslips. Briefly, EdU was dissolved in 0.9% sterile NaCl and filtered at 0.22 *μ*m, and was then added in medium 4 h before harvesting cells. Then cells were immediately fixed in 4% paraformaldehyde and conducted as instructions. Cell nucleus was counterstained with Hoechst 33342.

### Fluorescence, immunofluorescence and confocal microscopy

To study the function of EMC6 in inducing autophagosome formation, the GFP–LC3B-positive cells were detected by a confocal fluorescence microscope. GBM cell lines were plated on glass coverslips and then infected with Ad5–GFP–LC3B. After 20 h, cells were observed using fluorescence microscopy and imaged by a Leica SP2 confocal system (Leica Microsystems, Wetzlar, Hesse-Darmstadt, Germany). The number of GFP–LC3B puncta per cells was assessed in 10 non-overlapping fields, and statistical data were obtained from 3 independent experiments.

Brain tissue frozen sections were fixed with 4% formaldehyde, washed with PBS and blocked with PBS with 5% BSA and 0.25% Triton X-100 at room temperature for 1 h. The sections were incubated with indicated primary antibody at 4 °C overnight, washed with PBS three times and incubated with FITC-labeled secondary antibodies at 37 °C for 1 h. Nuclei were stained with Hoechst 33342 for 5 min. Finally, immunofluorescence was detected under a confocal fluorescence microscope (LSM 510 Meta plus Axiovert zoom; Carl Zeiss, Oberkochenza, Germany) with × 40/1.40 NA oil immersion objective lens (PlanApochromat; Carl Zeiss) and a camera (AxioCam HRm; Carl Zeiss). Images were processed and viewed using LSM Image Browser software. Sections of the brain tissues were stained in this study unless otherwise noted. Five randomly selected areas from each slide were examined for the rate of Ki67-positive cells or LC3B punctas under confocal fluorescence microscope by three independent, blinded investigators.

### Poly Q degradation assay

GBM cell lines were transfected with polyQ80-luciferase (or control polyQ19-luciferase) constructs. After 24 h, cells were lysed by passive lysis buffer from the DLR Assay System and centrifuged in a refrigerated centrifuge. The cleared lysates were transfered into a fresh tube and the firefly luciferase activity of polyQ80-luciferase or polyQ19-luciferase in each group was measured. polyQ19-luciferase was used as an internal control. Here, it was designed to measure only firefly luciferase reporter activity in the treated cell lysates. Briefly, 20 *μ*l of cell lysate prepared from polyQ80-luciferase or polyQ19-luciferase transfected cells were dispensed in triplicate into 96-well assay plate (Corning Incorporated COSTAR, NY, Washington, USA, 3925) pre-added into 100 *μ*l of luciferase assay buffer II (containing luciferase assay substrate) from the DLR Assay System according to the manufacturer's protocol. Then the assay plate was mixed by pipetting two or three times, and stabilized luminescent signal was measured by the Veritas Microplate Luminometer (Turner Biosystems, Bio-rad, Hercules, CA, USA). Data were expressed as the ratio of polyQ80-luciferase/polyQ19-luciferase luminescence signal values in each group as described previously.^23^ All samples were assayed in triplicate, and the results were shown with three independent experiments.

### Implant intracranial GBM in nude mice

A nude mouse intracranial model was established using 6–8-week-old female BALB/c nude mice (Experimental Animal Center, Peking University Health Sciences Center, Beijing, China). Mice were housed and maintained in a pathogen-free facility, and all experimental procedures and protocols were approved by the Institutional Authority for Laboratory Animal Care of Peking University. For *in vivo* treatments, EMC6-overexpressing U87 cells were injected into the brain of BALB/c nude mice in a total volume of 10 *μ*l (2 × 10^5^ cells), and tumors were monitored by luciferase signal every 7 days by *in vivo* Imaging System (IVIS) 100 (Caliper Life Science, Waltham, Massachusetts, USA) to monitor the development of GBM. Living Image software Version 4.3.1 (Caliper Life Science, Waltham, Massachusetts, USA) was used to acquire and quantify the bioluminescence imaging data sets. All animal experiments followed the National Institutes of Health guidelines for animal welfare. The luciferase signal curve and survival curve were drawn to study the function of EMC6-inhibiting GBM growth.

### Statistical analysis

Data are presented as the mean±S.D. Differences between groups were analyzed using the Student's *t*-test for continuous variables. Statistical significance in this study was set at *P*<0.05. All reported *P*-values are two-sided. All analyses were performed with GraphPad Prism 5 (GraphPad Software, Inc., La Jolla, CA, USA).

## Abbreviations

GBM: Glioblastoma
AKT: Protein kinase B (PKB)
EdU: 5-ethynyl-2′-deoxyuridine
EIF4EBP1: Eukaryotic translation initiation factor 4E-binding protein 1
EMC6: ER membrane protein complex subunit 6
mTOR: mammalian target of rapamycin
PIK3CA: phosphatidylinositol-4,5-bisphosphate 3-kinase, catalytic subunit alpha
RPS6KB1: ribosomal protein S6 kinases, polypeptide 1
SQSTM1: sequestosome-1
TMZ: Temozolomide

## References

[bib1] Ostrom QT, Gittleman H, Liao P, Rouse C, Chen Y, Dowling J et al. CBTRUS statistical report: primary brain and central nervous system tumors diagnosed in the United States in 2007-2011. Neuro Oncol 2014; 16 Suppl 4: iv1–63. 25304271 10.1093/neuonc/nou223 PMC4193675

[bib2] Van Meir EG, Hadjipanayis CG, Norden AD, Shu HK, Wen PY, Olson JJ. Exciting new advances in neuro-oncology: the avenue to a cure for malignant glioma. CA Cancer J Clin 2010; 60: 166–193. 20445000 10.3322/caac.20069 PMC2888474

[bib3] Reggiori F, Klionsky DJ. Autophagy in the eukaryotic cell. Eukaryot Cell 2002; 1: 11–21. 12455967 10.1128/EC.01.1.11-21.2002 PMC118053

[bib4] Mizushima N, Levine B. Autophagy in mammalian development and differentiation. Nat Cell Biol 2010; 12: 823–830. 20811354 10.1038/ncb0910-823 PMC3127249

[bib5] Rabinowitz JD, White E. Autophagy and metabolism. Science 2010; 330: 1344–1348. 21127245 10.1126/science.1193497 PMC3010857

[bib6] Maiuri MC, Kroemer G. Autophagy in stress and disease. Cell Death Differ 2015; 22: 365–366. 25661524 10.1038/cdd.2014.236 PMC4326584

[bib7] Morselli E, Galluzzi L, Kepp O, Vicencio JM, Criollo A, Maiuri MC et al. Anti- and pro-tumor functions of autophagy. Biochim Biophys Acta 2009; 1793: 1524–1532. 19371598 10.1016/j.bbamcr.2009.01.006

[bib8] Aredia F, Guaman Ortiz LM, Giansanti V, Scovassi AI. Autophagy and cancer. Cells 2012; 1: 520–534. 24710488 10.3390/cells1030520 PMC3901115

[bib9] Liu B, Wen X, Cheng Y. Survival or death: disequilibrating the oncogenic and tumor suppressive autophagy in cancer. Cell Death Dis 2013; 4: e892. 24176850 10.1038/cddis.2013.422 PMC3920945

[bib10] Cianfanelli V, D'Orazio M, Cecconi F. AMBRA1 and BECLIN 1 interplay in the crosstalk between autophagy and cell proliferation. Cell Cycle 2015; 14: 959–963. 25803737 10.1080/15384101.2015.1021526 PMC4615147

[bib11] Mathew R, Karp CM, Beaudoin B, Vuong N, Chen G, Chen HY et al. Autophagy suppresses tumorigenesis through elimination of p62. Cell 2009; 137: 1062–1075. 19524509 10.1016/j.cell.2009.03.048 PMC2802318

[bib12] Wei Y, Zou Z, Becker N, Anderson M, Sumpter R, Xiao G et al. EGFR-mediated Beclin 1 phosphorylation in autophagy suppression, tumor progression, and tumor chemoresistance. Cell 2013; 154: 1269–1284. 24034250 10.1016/j.cell.2013.08.015 PMC3917713

[bib13] Niu TK, Cheng Y, Ren X, Yang JM. Interaction of Beclin 1 with survivin regulates sensitivity of human glioma cells to TRAIL-induced apoptosis. FEBS Lett 2010; 584: 3519–3524. 20638385 10.1016/j.febslet.2010.07.018 PMC3210451

[bib14] Gao W, Shen Z, Shang L, Wang X. Upregulation of human autophagy-initiation kinase ULK1 by tumor suppressor p53 contributes to DNA-damage-induced cell death. Cell Death Differ 2011; 18: 1598–1607. 21475306 10.1038/cdd.2011.33 PMC3172118

[bib15] Graf MR, Jia W, Johnson RS, Dent P, Mitchell C, Loria RM. Autophagy and the functional roles of Atg5 and beclin-1 in the anti-tumor effects of 3beta androstene 17alpha diol neuro-steroid on malignant glioma cells. J Steroid Biochem Mol Biol 2009; 115: 137–145. 19375507 10.1016/j.jsbmb.2009.03.013

[bib16] Miracco C, Cosci E, Oliveri G, Luzi P, Pacenti L, Monciatti I et al. Protein and mRNA expression of autophagy gene Beclin 1 in human brain tumours. Int J Oncol 2007; 30: 429–436. 17203225

[bib17] Huang X, Bai HM, Chen L, Li B, Lu YC. Reduced expression of LC3B-II and Beclin 1 in glioblastoma multiforme indicates a down-regulated autophagic capacity that relates to the progression of astrocytic tumors. J Clin Neurosci 2010; 17: 1515–1519. 20863706 10.1016/j.jocn.2010.03.051

[bib18] Kaza N, Kohli L, Roth KA. Autophagy in brain tumors: a new target for therapeutic intervention. Brain Pathol 2012; 22: 89–98. 22150924 10.1111/j.1750-3639.2011.00544.x PMC3243074

[bib19] Zhuang W, Qin Z, Liang Z. The role of autophagy in sensitizing malignant glioma cells to radiation therapy. Acta Biochim Biophys Sin (Shanghai) 2009; 41: 341–351. 19430698 10.1093/abbs/gmp028

[bib20] Noack J, Choi J, Richter K, Kopp-Schneider A, Regnier-Vigouroux A. A sphingosine kinase inhibitor combined with temozolomide induces glioblastoma cell death through accumulation of dihydrosphingosine and dihydroceramide, endoplasmic reticulum stress and autophagy. Cell Death Dis 2014; 5: e1425. 25255218 10.1038/cddis.2014.384 PMC4540206

[bib21] Kanzawa T, Germano IM, Komata T, Ito H, Kondo Y, Kondo S. Role of autophagy in temozolomide-induced cytotoxicity for malignant glioma cells. Cell Death Differ 2004; 11: 448–457. 14713959 10.1038/sj.cdd.4401359

[bib22] Chiu HW, Ho YS, Wang YJ. Arsenic trioxide induces autophagy and apoptosis in human glioma cells *in vitro* and *in vivo* through downregulation of survivin. J Mol Med (Berl) 2011; 89: 927–941. 21594580 10.1007/s00109-011-0763-1

[bib23] Li Y, Zhao Y, Hu J, Xiao J, Qu L, Wang Z et al. A novel ER-localized transmembrane protein, EMC6, interacts with RAB5A and regulates cell autophagy. Autophagy 2013; 9: 150–163. 23182941 10.4161/auto.22742 PMC3552880

[bib24] Yang Z, Klionsky DJ. Mammalian autophagy: core molecular machinery and signaling regulation. Curr Opin Cell Biol 2010; 22: 124–131. 20034776 10.1016/j.ceb.2009.11.014 PMC2854249

[bib25] Vogt PK. PI 3-kinase, mTOR, protein synthesis and cancer. Trends Mol Med 2001; 7: 482–484. 11689313 10.1016/s1471-4914(01)02161-x

[bib26] Surawicz TS, Davis F, Freels S, Laws ER Jr, Menck HR. Brain tumor survival: results from the National Cancer Data Base. J Neurooncol 1998; 40: 151–160. 9892097 10.1023/a:1006091608586

[bib27] Sekulic A, Hudson CC, Homme JL, Yin P, Otterness DM, Karnitz LM et al. A direct linkage between the phosphoinositide 3-kinase-AKT signaling pathway and the mammalian target of rapamycin in mitogen-stimulated and transformed cells. Cancer Res 2000; 60: 3504–3513. 10910062

[bib28] Matsunaga K, Morita E, Saitoh T, Akira S, Ktistakis NT, Izumi T et al. Autophagy requires endoplasmic reticulum targeting of the PI3-kinase complex via Atg14L. J Cell Biol 2010; 190: 511–521. 20713597 10.1083/jcb.200911141 PMC2928018

[bib29] Misra S, Miller GJ, Hurley JH. Recognizing phosphatidylinositol 3-phosphate. Cell 2001; 107: 559–562. 11733055 10.1016/s0092-8674(01)00594-3

[bib30] Friedman HS, Kerby T, Calvert H. Temozolomide and treatment of malignant glioma. Clin Cancer Res 2000; 6: 2585–2597. 10914698

[bib31] Gerdes J, Schwab U, Lemke H, Stein H. Production of a mouse monoclonal antibody reactive with a human nuclear antigen associated with cell proliferation. Int J Cancer 1983; 31: 13–20. 6339421 10.1002/ijc.2910310104

[bib32] Yuan JP, Wang LW, Qu AP, Chen JM, Xiang QM, Chen C et al. Quantum dots-based quantitative and *in situ* multiple imaging on ki67 and cytokeratin to improve ki67 assessment in breast cancer. PLoS One 2015; 10: e0122734. 25856425 10.1371/journal.pone.0122734 PMC4391934

[bib33] Ponnala S, Veeravalli KK, Chetty C, Dinh DH, Rao JS. Regulation of DNA repair mechanism in human glioma xenograft cells both *in vitro* and *in vivo* in nude mice. PLoS One 2011; 6: e26191. 22022560 10.1371/journal.pone.0026191 PMC3193530

[bib34] Bjorkoy G, Lamark T, Johansen T. p62/SQSTM1: a missing link between protein aggregates and the autophagy machinery. Autophagy 2006; 2: 138–139. 16874037 10.4161/auto.2.2.2405

[bib35] Burdelski C, Reiswig V, Hube-Magg C, Kluth M, Minner S, Koop C et al. Cytoplasmic accumulation of Sequestosome 1 (p62) is a predictor of biochemical recurrence, rapid tumor cell proliferation and genomic instability in prostate cancer. Clin Cancer Res 2015; 21: 3471–3479. 25925890 10.1158/1078-0432.CCR-14-0620

[bib36] Laplante M, Sabatini DM. mTOR signaling in growth control and disease. Cell 2012; 149: 274–293. 22500797 10.1016/j.cell.2012.03.017 PMC3331679

[bib37] Vivanco I, Sawyers CL. The phosphatidylinositol 3-Kinase AKT pathway in human cancer. Nat Rev Cancer 2002; 2: 489–501. 12094235 10.1038/nrc839

[bib38] Takeuchi H, Kondo Y, Fujiwara K, Kanzawa T, Aoki H, Mills GB et al. Synergistic augmentation of rapamycin-induced autophagy in malignant glioma cells by phosphatidylinositol 3-kinase/protein kinase B inhibitors. Cancer Res 2005; 65: 3336–3346. 15833867 10.1158/0008-5472.CAN-04-3640

[bib39] Dowling RJ, Topisirovic I, Alain T, Bidinosti M, Fonseca BD, Petroulakis E et al. mTORC1-mediated cell proliferation, but not cell growth, controlled by the 4E-BPs. Science 2010; 328: 1172–1176. 20508131 10.1126/science.1187532 PMC2893390

[bib40] Hsieh AC, Costa M, Zollo O, Davis C, Feldman ME, Testa JR et al. Genetic dissection of the oncogenic mTOR pathway reveals druggable addiction to translational control via 4EBP-eIF4E. Cancer Cell 2010; 17: 249–261. 20227039 10.1016/j.ccr.2010.01.021 PMC2901095

[bib41] Filippi-Chiela EC, Thome MP, Bueno e Silva MM, Pelegrini AL, Ledur PF, Garicochea B et al. Resveratrol abrogates the temozolomide-induced G2 arrest leading to mitotic catastrophe and reinforces the temozolomide-induced senescence in glioma cells. BMC Cancer 2013; 13: 147. 23522185 10.1186/1471-2407-13-147 PMC3635906

[bib42] Kanzawa T et al. Role of autophagy in temozolomide-induced cytotoxicity for malignant glioma cells. Cell Death Differ 2004; 11: 448–457. 14713959 10.1038/sj.cdd.4401359

[bib43] Koekkoek JA, Dirven L, Heimans JJ, Postma TJ, Vos MJ, Reijneveld JC et al. Seizure reduction in a low-grade glioma: more than a beneficial side effect of temozolomide. J Neurol Neurosurg Psychiatry 2015; 86: 366–373. 25055819 10.1136/jnnp-2014-308136

