# Supplementary material for: EMC6/TMEM93 suppresses glioblastoma proliferation by modulating autophagy
Source: Cell Death Dis. 2016 Jan 14;7(1):e2043–. doi: 10.1038/cddis.2015.408 (PMC4816184; doi:10.1038/cddis.2015.408)
Supplement: Supplementary Information [file cddis2015408x1.doc]

**Supplementary data**

***EMC6/TMEM93* suppresses glioblastoma proliferation by modulating autophagy**

Xue Shen ^1^, Shifeng Kan ^1^, Jia Hu ^2,3^, Mengtao Li ^1^, Guang Lu ^1,4^, Mingzhi Zhang ^1^, Shu Zhang ^5^, Yuelong Hou ^1^, Yingyu Chen ^2,3,*^, Yun Bai ^1,*^

^1^ Department of Cell Biology, School of Basic Medical Sciences, Peking University Health Science Center, Beijing 100191, China

^2^ Key Laboratory of Medical Immunology, Ministry of Health, Peking University Health Science Center, Beijing 100191, China

^3^ Peking University Center for Human Disease Genomics, Peking University, Beijing 100191, China

^4^ Department of Physiology, Yong Loo Lin School of Medicine, National University of Singapore, Singapore 117597

^5^ Department of Cardiology, Peking University Third Hospital, Beijing 100191, China

*Corresponding authors. E-mail addresses: baiyun@bjmu.edu.cn (Y. Bai) or yingyu_chen@bjmu.edu.cn (Y. Chen)

Phone: +86-10-82801495 (Y. Bai) or +86-10-82801149 (Y. Chen).

**Materials and Methods**

**Cell Transfection**

Three chemically synthesized EMC6 siRNA and negative control siRNA were used for transfection. The sequences of EMC6 siRNA-1 were: sense strand 5'-UGUUCUGGACGUUCCUCUATT-3'; EMC6 siRNA-2 were: sense strand 5'-GGAAGGAGGUGGAACAAAUTT-3'; EMC6 siRNA-3 were: sense strand 5'-GCCUCUACGGCUUCAUCUUTT-3'. To make the transfection mixture, solutions A and B were first prepared in 1.5 ml microcentrifuge tubes. Solution A was 250 μl Opti-MEM medium mixed with 10μl EMC6 siRNA or negative control siRNA. Then solution B was 250 μl Opti-MEM medium was mixed with 5μl Lipofectamine2000 (Invitrogen, Carlsbad, CA, USA). Then both solutions were placed at room temperature for 5min. Then solution A was mixed with solution B and allowed to stay at room temperature for 20min. U87 cells in 6-well plates were washed with PBS twice and then the transfection mixture was added to the cells. The plates were gently rocked to make sure all cells came into contact with the mixture, and then 1 ml medium was added to the cells.

**Real-time PCR**

Real-time PCR was carried out using SYBR green Real time PCR kit from Qiagen on an Eppendorf mastercycler, eprealplex (Eppendorf, Germany). Relative mRNA expression was determined by normalization to the expression of a housekeeping gene, GAPDH gene expression. Primer list is provided below: CAACTGTTACAATCAAGGTCTATGAAG and CAAAGGTGACTTCAATCTGTGG for GRP78; CCAAGGGAGAACCAGGAAACG and TCACCATTCGGTCAATCAGAGC for CHOP; CTGGATGAAGATTGGGATT and TGACCGAGGAGACGAGAC for ATF6; ATGACCGAAATGAGCTTCCTG and GCTGGAGAACCCATGAGGT for ATF4; AAGGCGCTTACAGCTCAATG and CTGGGAGGCATAGACCATGT for LC3B; AAAGATGTGCTTCGAGATGTGT and CACTTTGTCAGTTACCAACGTCA for ATG5; TAGAGCGAACACGAACCATCC and CACTGCCAAAACACTCATAGAGA for ATG12; GAAGGTGAAGGTCGGAGTC and GAAGATGGTGATGGGATTTC were used for GAPDH.

**Flow cytometer.** Apoptosis levels were detected by FITC–Annexin V staining Detection kit (Beijing Biosea Biotechnology Co., Ltd., Beijing, China) with flow cytometry. Briefly, cells in six-well plates in triplicate were trypsinized and resuspended in 100 ul binding buffer (10 mM HEPES, pH 7.4, 140 mM NaCl, 1 mM MgCl2, 5 mM KCl, 2.5 mM CaCl2) containing 5ul FITC-conjugated Annexin V, incubated at room temperature in the dark for 30 min. Then, the cells were harvested and analyzed on a FACSCalibur flow cytometer.

**Protein extraction and Western blotting.** Total proteins from cells were extracted with radio immunoprecipitation assay buffer (50 mM Tris, pH 7.4, 150 mM NaCl, 1% Triton X-100, 1% sodium deoxycholate, 0.1% SDS, and with freshly added proteinase inhibitor cocktail and phosphatase inhibitors). Protein concentrations were determined by the BCA protein assay reagent (NCI3227CH, Pierce). Equal amounts of proteins were separated by SDS-PAGE electrophoresis and transferred to nitrocellulose membranes. After blocking with 5% nonfat milk for 1 h at room temperature, the membranes were incubated overnight at 4°C with primary antibodies, and then the secondary antibodies. The membranes were then washed with PBS containing 0.1% Tween 20 and scanned with the Odyssey Infrared Imaging System (LI-COR Biosciences, Lincoln, NE, USA) by setting the detection channels at 700 nm (for Alexa Fluor 680) and 800 nm (for IRDye 800CW). Scanned bands were quantified using the Image J software and normalized against the protein level of β-actin/ACTB. Results are representative of at least three experiments.

Previous treatment before Western blotting: CQ treated cells with or without EMC6-overexpression (or *EMC6*-knockdown) at 25 μM for 2 h before harvesting cells. Perifosine treated cells with or without EMC6-overexpression (or *EMC6*-knockdown) at 30 μM for 16 h before harvesting cells. And 3-MA treated cells with or without EMC6-overexpression (or *EMC6*-knockdown) at 5 μM for 4 h before harvesting cells.

**Implant Intracranial GBM in nude mice.** A nude mouse intracranial model was established using 6–8 week-old female BALB/c nude mice (Experimental Animal Center, Peking University Health Sciences Center, Beijing, China). Mice were housed and maintained in a pathogen-free facility, and all experimental procedures and protocols were approved by the Institutional Authority for Laboratory Animal Care of Peking University. For in vivo treatments, *EMC6*-knockdown U87 cells were injected into the intracranial brain of BALB/c nude mice in a total volume of 10μl (2×10^5^ cells), and tumor were monitored by luciferase signal every 7 days. The luciferase signal curve and survival curve was drawn to study the function of EMC6 inhibiting GBM growth.

**TUNEL Assay.** An ApopTag Peroxidase *In Situ* Apoptosis Detection Kit (Chemicon International, Temecula, CA) was used for TUNEL staining. The brain frozen sections were incubated with 3% hydrogen peroxide for 5min at room temperature to quench endogenous peroxidase activity. Next, the sections were sequentially incubated with TdT enzyme for 1 h at 37°C. The nuclei were stained with Hoechst 33342.

**Statistical analysis.** Data are presented as the mean ± SD. Differences between groups were analyzed using the Student t test for continuous variables. Statistical significance in this study was set at p < 0.05. All reported p values are two-sided. All analyses were performed with GraphPad Prism 5.

**Supplementary figure legends**

**Supplementary Figure 1. Overexpression of EMC6 does not induce significant apoptosis in GBM cells. (a)** Cell apoptosis of SHG44, U87 and U251 cell lines with or without of EMC6-overexpression was detected by flow cytometry. **(b)** The number of apoptotic cells was calculated. Each bar represents the mean ± SD from three independent experiments.

**Supplementary Figure 2. Knockdown of *EMC6* fails to induce significant apoptosis in glioma cells. (a)** Cell apoptosis of SHG44 and U87 cell lines with or without of *EMC6*- Knockdown was detected by flow cytometry. **(b)** The number of apoptotic cells was calculated. Each bar represents the mean ± SD from three independent experiments.

**Supplementary Figure 3. EMC6 promotes autophagy in GBM cells.** **(a and b)** The levels of LC3B-II in SHG44, U87 and U251 cells with or without EMC6-overexpression were detected by western blot. Histogram shows the quantification of the ratio of the LC3B-II/ACTB. The average value in mock group was normalized as 1. Data are means ± SD of results from three independent experiments. ***P* < 0.01. **(c and d)** SHG44 and U251 cells with or without EMC6-overexpression were treated in the presence or absence with CQ (25 μM) for 2 h. The levels of LC3B-II were detected by western blot. Histogram shows the quantification of the ratio of the LC3B-II/ACTB. The average value in vector group without CQ treatment was normalized as 1. Data are means ± SD of results from three independent experiments. ***P* < 0.01. **(e and f)** The levels of LC3B-II in SHG44 and U87 cells with or without *EMC6*-knockdown were detected by western blot. Histogram shows the quantification of the ratio of the LC3B-II/ACTB. The average value in mock group was normalized as 1. Data are means ± SD of results from three independent experiments. **P* < 0.05, ***P* < 0.01. **(g and h)** SHG44 cells with or without *EMC6*-knockdown were treated with or without CQ (25 μM) for 2 h. The levels of LC3B-II were detected by western blot. Histogram shows the quantification of the ratio of the LC3B-II/ACTB. The average value in shRNA vector group without CQ treatment was normalized as 1. Data are means ± SD of results from three independent experiments. **P* < 0.05, ***P* < 0.01. **(i)** U87 cells with or without EMC6-overexpression or *EMC6*-knockdown were infected with Ad5-GFP–LC3 at 50 MOI for 20 h. The indicated cells were lysed and subjected to immunoblot analysis using the GFP antibodies. ACTB was detected as the protein loading control. **(j)** Histogram shows the quantification of the ratio of the GFP/ACTB of **(i)**. The average value in vector group was normalized as 1. Data are means ± SD of results from three independent experiments. *P < 0.05, ***P < 0.001. **(k)** Expression of LC3B-II in U87 cells after EMC6-overexpression at 0, 12, 24, 36h was analyzed by Western blot. **(l)** Quantification of LCB3-II levels relative to ACTB in cells treated as in **(k)**. **(m)** U87 cells apoptosis after EMC6-overexpression at 0, 12, 24, 36h was analyzed by Flow cytometry. **(n)** Quantification of cells apoptosis treated as in (m).

**Supplementary Figure 4. EMC6-knockdown attenuates cell autophagy in U87 cells. (a)** The mRNA levels of EMC6 in U87 cells transfected with indicated siRNA were detected by Real-time PCR. ***p < 0.001. **(b)** Expression of EMC6 in U87 cells transfected with indicated siRNA was analyzed by Western blot. **(c)** Quantification of EMC6 levels relative to ACTB in cells treated as in **(b)**. **(d)** U87 cells were plated in glass slides, transfected with indicated siRNA and incorporated with EdU. 4 h later, the indicated cells were performed by immunofluorescence assay. Percentage of EdU positive cells in total indicated cells were shown from five randomly selected areas from each slide. Each bar represents the mean ± SD from three independent experiments. *p < 0.05. (e) U87 cells U87 cells were transfected with indicated siRNA, treated with the CQ (25 μM) for 2 h. The levels of LC3B-II and SQSTM1 were detected by western blot. **(f)** Quantification of LC3B-II and SQSTM1 levels relative to ACTB in cells treated as in **(e)**. Average value in control cells was normalized as 1. Data are means ± SD of results from 3 experiments, ***P < 0.001.

**Supplementary Figure 5. EMC6 induces autophagy through the PIK3CA/AKT/mTOR pathway. (a)** U87 cells with or without EMC6-overexpression were transfected with RHEB-Q64L or RHEB-D60K Vector. The levels of p-RPS6KB1, LC3B-II and SQSTM1 were detected by western blot. **(b)** Quantification of LC3B-II levels relative to ACTB and p-RPS6KB1 to its total protein in cells treated as in **(a)**. Average value in vector group was normalized as 1. Data are means ± SD of results from 3 experiments, ***P* < 0.01, n.s, not significance. **(c)** U87 cells with or without *EMC6*-knockdown were treated in the present or absent 20 μM Perifosine for 16 h and harvested to conduct immunoblot analysis using the indicated antibodies. **(d)** Histogram shows the quantification of the ratio of the relative proteins/ ACTB of **(c)**. The average value in shRNA vector group was normalized as 1. Data are means ± SD of results from three independent experiments. **P* < 0.05, ***P* < 0.01. **(e)** U87 cells with or without EMC6-overexpression were treated with CQ (25 μM for 2h) and 3-MA (5 μM for 4h) or not, and harvested to conduct immunoblot analysis using the indicated antibodies. **(f)** Histogram shows the quantification of the ratio of the relative proteins/ ACTB of **(e)**. The average value in only 3-MA and CQ-treated cells was normalized as 1. Data are means ± SD of results from three independent experiments. **P* < 0.05, ****P* < 0.001.

**Supplementary Figure 6. EMC6 induces autophagy is not associated with the ER stress. (a, b and c)** The mRNA levels of *ATF4, CHOP, ATF6, GRT78, ATG5, ATG6* and *LC3B* in the three GMB cells with or without EMC6-overexpression were detected by Real-time PCR. Relative amount of indicated mRNA levels relative to GAPDH in cells with or without EMC6-overexpression. Average value in vector group was normalized as 1. Data are means ± SD of results from 3 experiments, ****P* < 0.001. **(d and e)** The mRNA levels of *ATF4, CHOP, ATF6, GRT78, ATG5, ATG6* and *LC3B* in cells with or without *EMC6*-knockdown were detected by Real-time PCR. Relative amount of indicated mRNA levels relative to GAPDH in cells with or without EMC6-overexpression. Average value in shRNA vector group was normalized as 1. Data are means ± SD of results from 3 experiments, ****P* < 0.001.

**Supplementary Figure 7. *EMC6* knockdown promotes the development of GBM in vivo. (a)** The U87 cells with or without *EMC6*-knockdown stably expressing luciferase were injected into intracranial brain of BALB/c nude mice. Luciferase signals in different treatment groups were detected every 7 days by in vivo bioluminescence imaging (IVIS 100 Imaging System) to monitor the development of glioblastoma. n=6. Living Image software Version 4.3.1 was used to acquire and quantify the bioluminescence imaging data sets. **(b)** The survival days differences between the indicated groups of mice were shown by survival curve until all mice were dead. n=6. **P < 0.01.
