# Supplementary figures and images for: EMC6/TMEM93 suppresses glioblastoma proliferation by modulating autophagy
Source: Cell Death Dis. 2016 Jan 14;7(1):e2043–. doi: 10.1038/cddis.2015.408 (PMC4816184; doi:10.1038/cddis.2015.408)

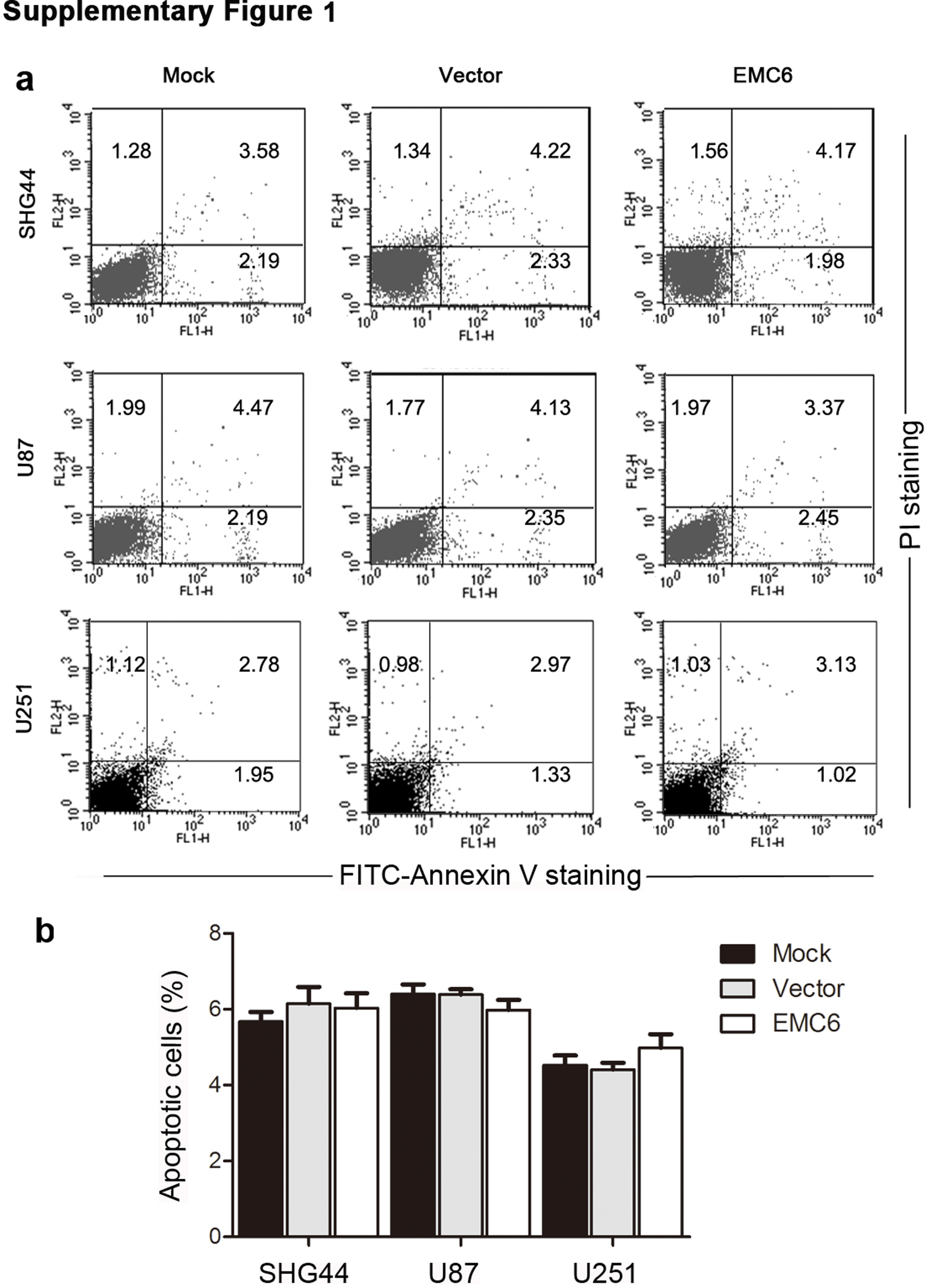

Supplement: Supplementary Figure 1 [file cddis2015408x2.tif]

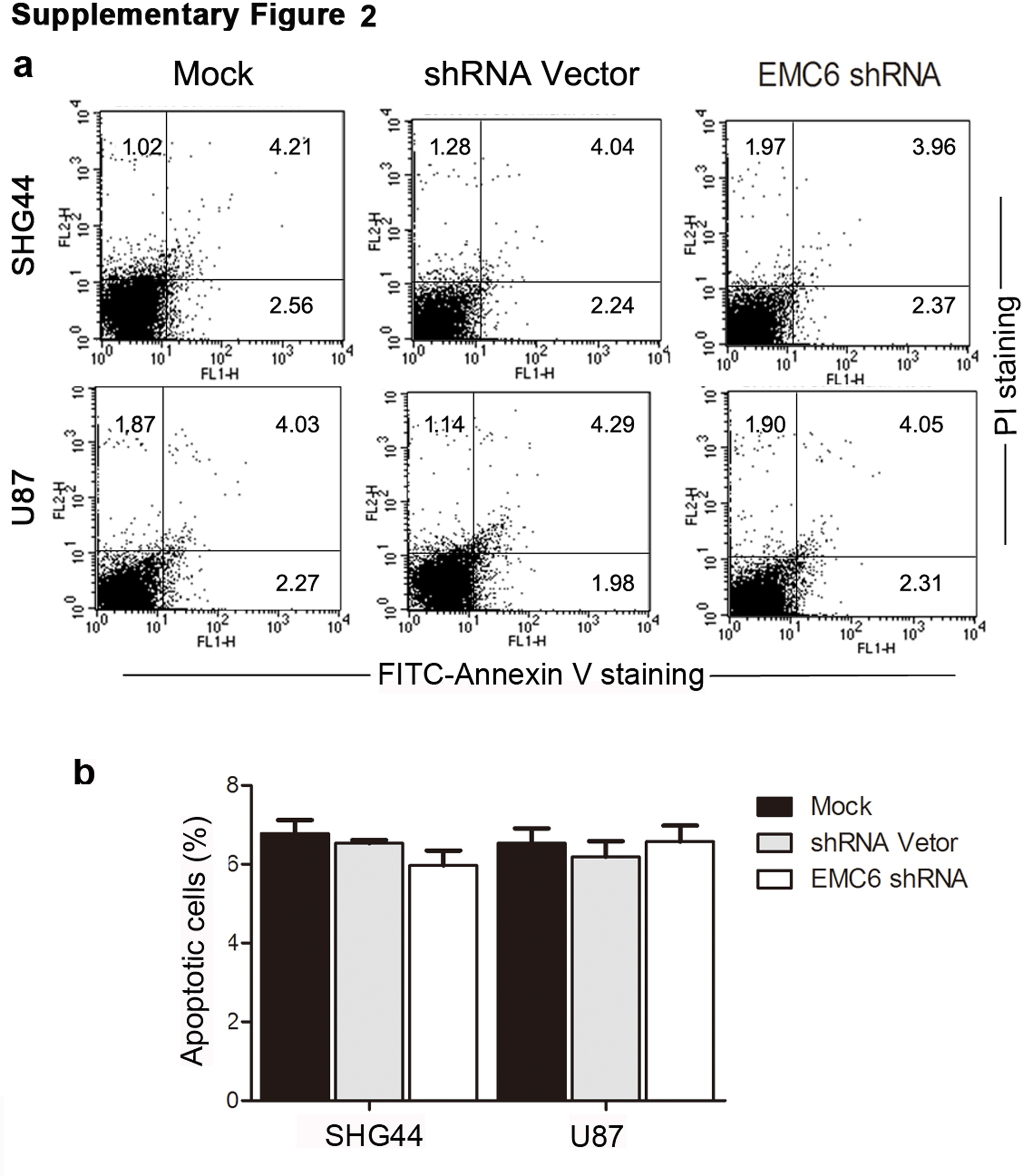

Supplement: Supplementary Figure 2 [file cddis2015408x3.tif]

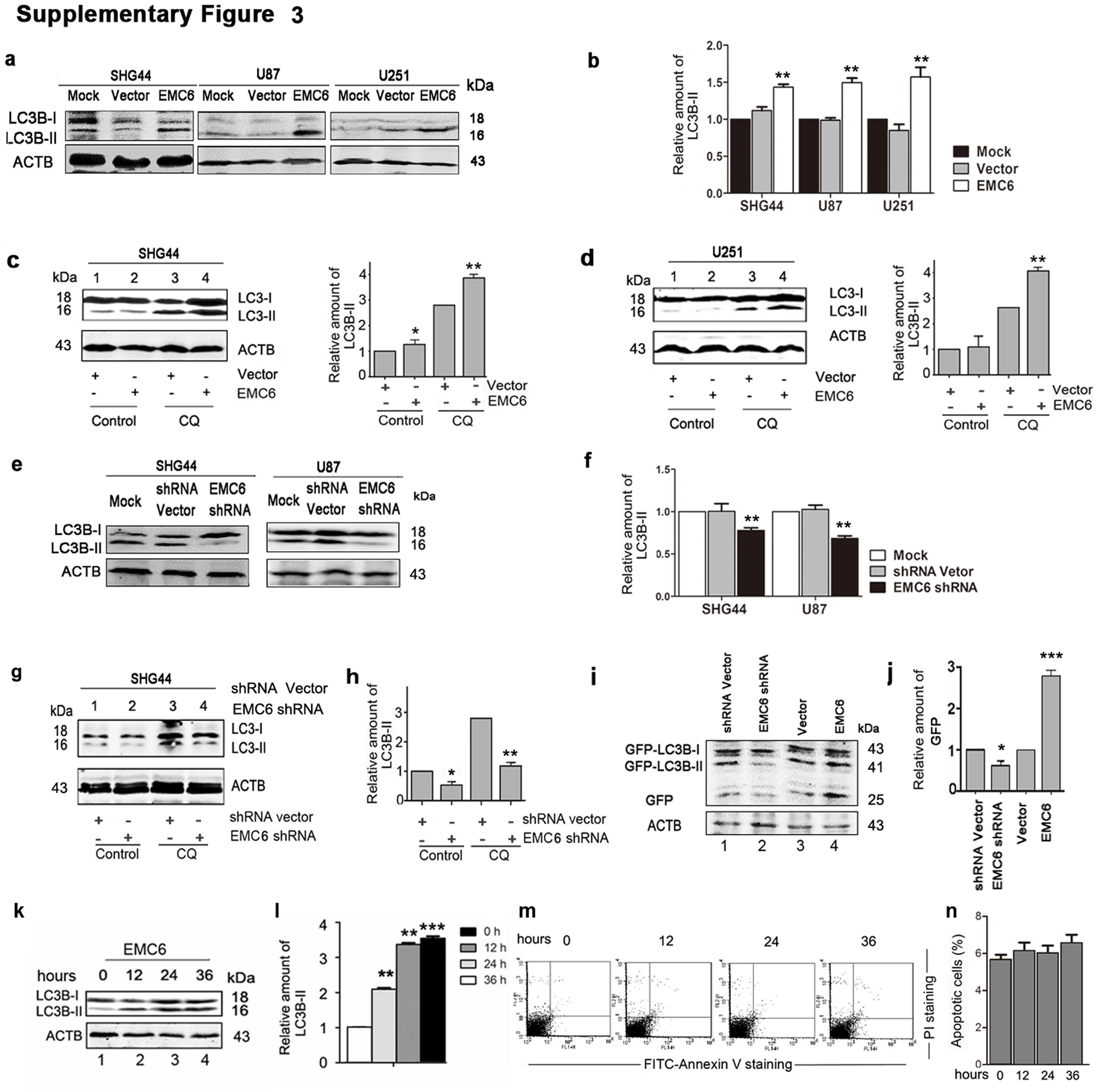

Supplement: Supplementary Figure 3 [file cddis2015408x4.tif]

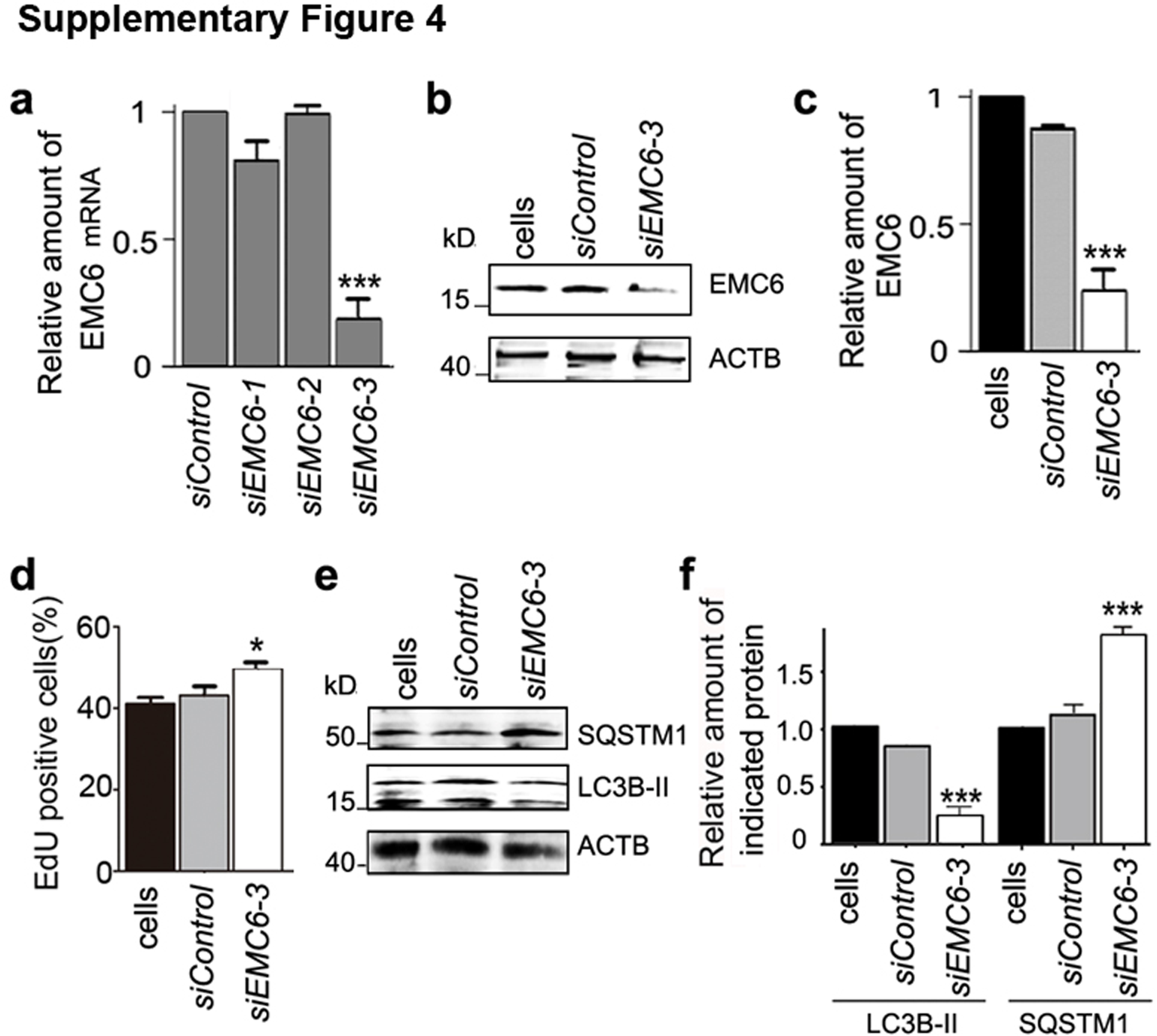

Supplement: Supplementary Figure 4 [file cddis2015408x5.tif]

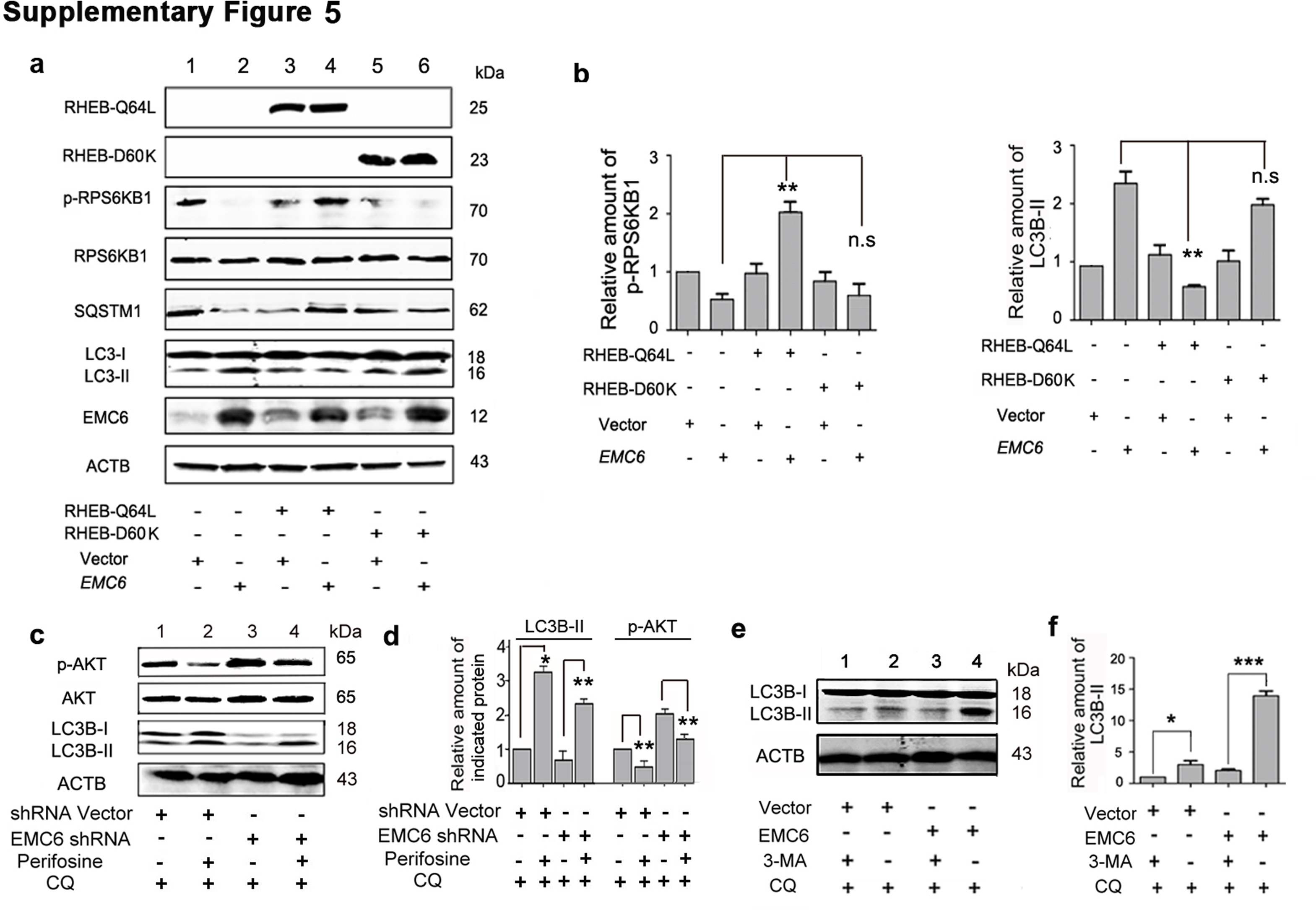

Supplement: Supplementary Figure 5 [file cddis2015408x6.tif]

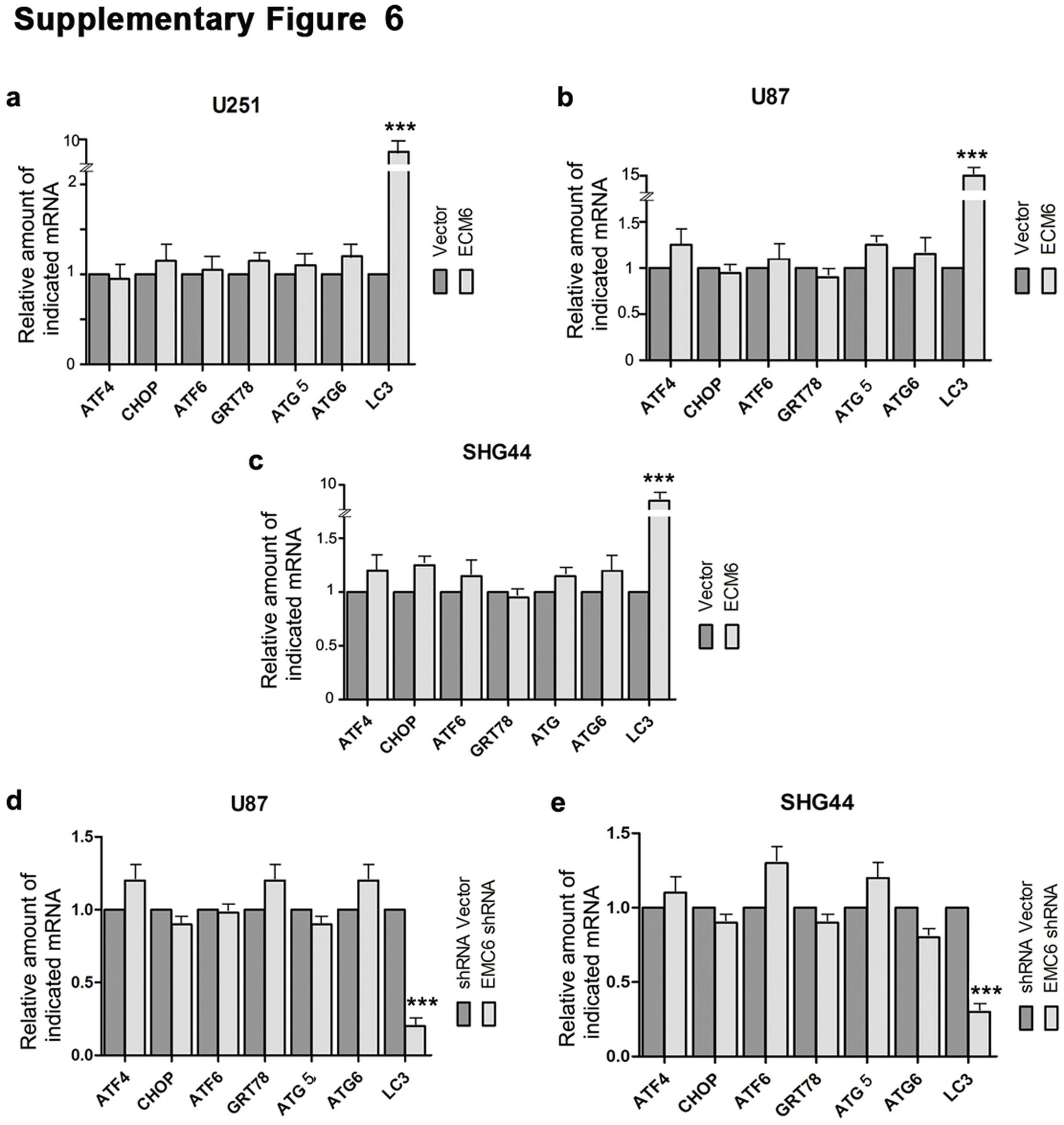

Supplement: Supplementary Figure 6 [file cddis2015408x7.tif]

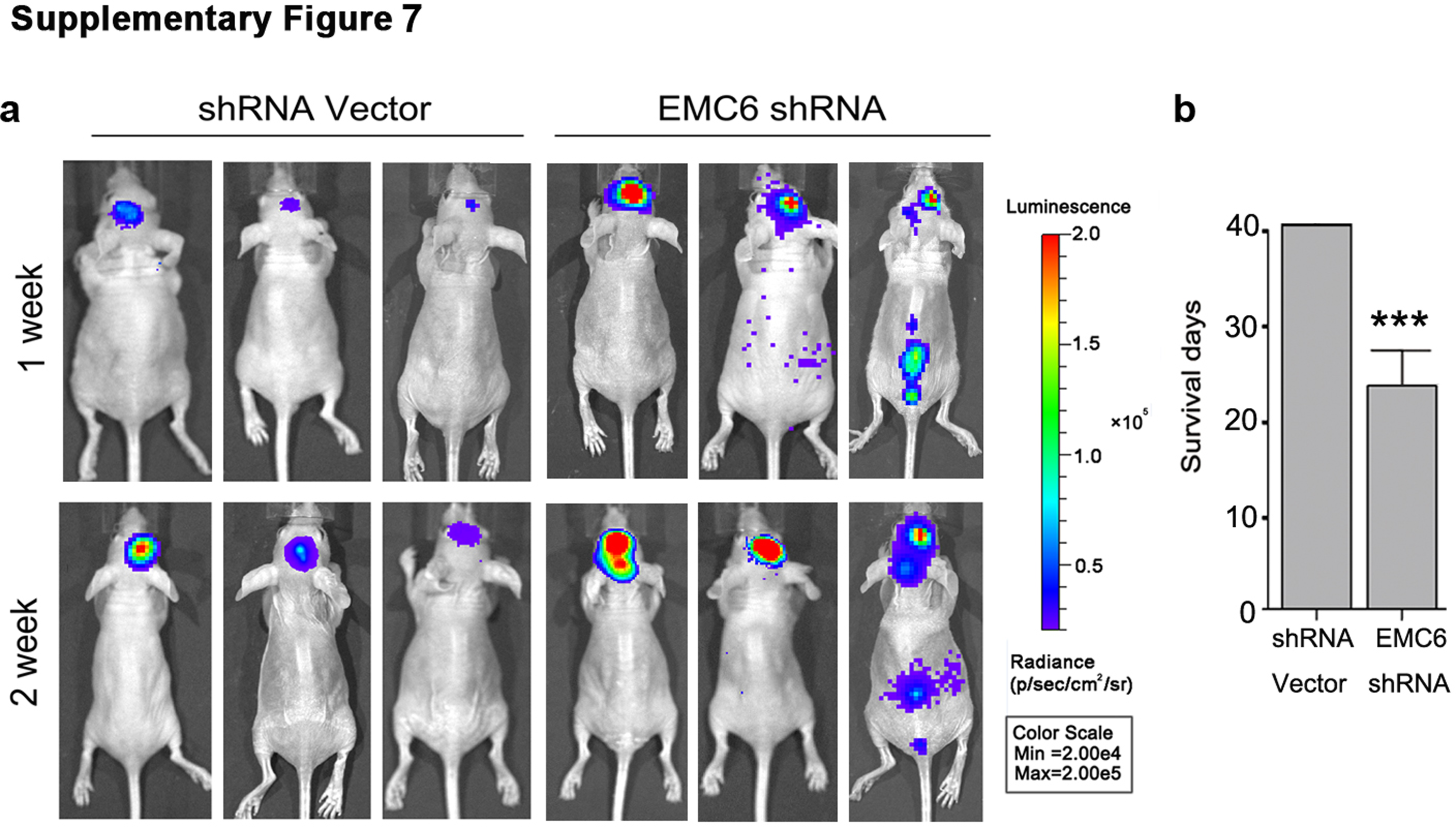

Supplement: Supplementary Figure 7 [file cddis2015408x8.tif]
